# To use financial incentives or not? Insights from experiments in encouraging sanitation investments in four countries

**DOI:** 10.1016/j.worlddev.2024.106791

**Published:** 2025-03

**Authors:** Sanghmitra Gautam, Michael Gechter, Raymond P. Guiteras, Ahmed Mushfiq Mobarak

**Affiliations:** a Department of Economics, Ca’ Foscari University of Venice, Italy; b Sloan School of Business, MIT, United States of America; c Harvard Kennedy School, United States of America; d North Carolina State University, United States of America; e Yale University, United States of America

**Keywords:** Sanitation, Impact evaluation

## Abstract

We conduct a systematic re-analysis of intervention-based studies that promote hygienic latrines and evaluate via experimental methods. We impose systematic inclusion criteria to identify such studies and compile their microdata to harmonize outcome measures, covariates, and estimands across studies. We then re-analyze their data to report metrics that are consistently defined and measured across studies. We compare the relative effectiveness of different classes of interventions implemented in overlapping ways across four countries: community-level demand encouragement, sanitation subsidies, product information campaigns, and microcredit to finance product purchases. In the sample of studies meeting our inclusion criteria, interventions that offer financial benefits generally outperform information and education campaigns in increasing adoption of improved sanitation. Contrary to a policy concern about sustainability, financial incentives do not undermine usage of adopted latrines. Effects vary by share of women in the household, in both positive and negative directions, and differ little by poverty status.

## Highlights

• We conduct a systematic re-analysis of studies of improved sanitation adoption.
• We include RCTs with publicly available micro-data including adoption at baseline.
• We harmonize data across studies for consistent outcomes, covariates and estimands.
• We compare the effectiveness of different intervention types across four countries.
• In our sample, financial incentives generally outperform information and motivation.
• Effects vary by share of women in the household, but differ little by poverty status.

## 1 Introduction

The United Nations Sustainable Development Goals (SDGs) acknowledge access to clean water and sanitation as fundamental human rights. Over 400 million people, or about 5% of the world’s population, practiced open defecation in 2022, and another 545 million did not have access to an improved sanitation facility (WHO/UNICEF, 2023). Diarrheal disease attributable to inadequate sanitation is estimated to cause 560,000 deaths and the loss of nearly 30 million disability-adjusted life years annually (Wolf et al., 2023). Better sanitation improves children’s physical growth (Fuller, Villamor, Cevallos, Trostle, & Eisenberg, 2016) and cognitive development (Spears & Lamba, 2016). Beyond disease, the lack of sanitation facilities forces women out in the dark in rural areas, which could increase the risk of sexual assault (Hossain, Mahajan, & Sekhri, 2022).

Simple technologies like pour-flush latrines have the potential to reduce the prevalence of open defecation, but such technologies need to be widely deployed and adopted. Large government-led sanitation programs in India focused on toilet construction to address supply deficiencies (Spears & Lamba, 2016). The most recent iteration – the Swatch Bharat Mission – (SBM) initially engaged in efforts to promote demand-side behavior change, but a process evaluation shows that ultimately SBM’s goals were translated largely into achieving toilet construction targets (Munoz Boudet et al., 2023).

In contrast, an intervention-based academic literature on sanitation promotion, often evaluating NGO-led programs, displays a much heavier tilt towards demand-side drivers. That literature focuses more on individual or community-level behavior change. Academic studies have examined social influence in demand Guiteras, Levinsohn, and Mobarak (2015), cultural practices and beliefs (Coffey et al., 2014), microcredit for toilet investments (BenYishay et al., 2017), social penalties for non-investment (Stopnitzky, 2017), education and motivation to help communities overcome informational constraints (Pickering, Djebbari, Lopez, Coulibaly, & Alzua, 2015), welfare impacts of sanitation policies in the presence of externalities (Gautam, 2023), and joint investment commitments by neighbors given the large negative public health externalities from open defecation (Bakhtiar, Guiteras, Levinsohn, & Mobarak, 2023).

This paper aims to conduct a systematic re-analysis of intervention-based studies that promoted improved sanitation and used Randomized Controlled Trials (RCTs) to rigorously evaluate their strategies. We first impose a systematic inclusion criterion to identify such studies, then compile their data in order to harmonize outcome and covariate measures as well as estimands across studies, and finally we re-analyze their data to report metrics that are consistently defined and measured across studies. We then summarize the key insights from that literature based on our re-analysis, which allows us to compare the relative effectiveness of different classes of interventions – such as providing sanitation subsidies, conducting information campaigns, or offering microcredit to poor households – that were implemented across four countries.

This approach allows us to address an important policy question that has animated many global debates about sanitation promotion: the relevance of financial subsidies in behavior change campaigns. The proponents of *Community-Led Total Sanitation* (CLTS) – by far the most popular behavioral intervention in this sector (Bardosh, 2015) – are often vehemently against subsidies for toilet construction, because subsidies are thought to undermine a community’s intrinsic motivation and CLTS scaling potential, or create dependence (Kar & Chambers, 2008). USAID WASHPaLS (2018) writes, “avoidance of subsidies is perhaps the most fundamental tenet of CLTS”. This view has important policy implications because CLTS is described as a “revolutionary idea” that has spread rapidly across 29 developing countries in Africa and Asia by 2018 from its origins in Bangladesh in 1999 (USAID WASHPaLS, 2018). But this anti-subsidy view stands in contrast to a common finding in the development economics literature, that price is an important deterrent to investments in technologies with the potential to improve population health (Bates, Glennerster, Gumede, & Duflo, 2012). Financial barriers have been identified as an impediment for a wider class of technologies and products that can address important development challenges, such as drinking water disinfectants (Ashraf, Berry, & Shapiro, 2010), insecticide-treated bednets (Tarozzi, Mahajan, Blackburn, Kopf, Krishnan et al., 2014), and improved cookstoves (Mobarak, Dwivedi, Bailis, Hildemann, & Miller, 2012).

To construct our sample of studies for re-analysis, we applied the following inclusion criteria. First, studies must focus on the adoption of sanitation products rather than increasing the use of already-owned products. Second, studies must use a randomized research design to abstract from issues of imperfect compliance and must have microdata available so that we can harmonize outcome and covariate values. We search for studies matching our criteria by applying reference tracking, also known as forward and backward snowballing, to three prominent meta-analyses: Cameron, Gertler, Shah, Alzua, Martinez et al. (2022), Garn et al. (2017), and Whittington, Radin, and Jeuland (2020). This process identified over one hundred candidate studies, four of which met all of our inclusion criteria: Guiteras et al. (2015) in Bangladesh, Cameron, Shah, and Olivia (2013) in Indonesia, BenYishay et al. (2017) in Cambodia, and Patil et al. (2014) in India. Our review, therefore, covers some of the most populous developing countries in the world. These countries are even more relevant in the sanitation sector specifically because their populations represented the majority of open defecators in the world during the periods when those studies were conducted.

These studies experimented with an overlapping set of interventions. For example, three of the four published studies conducted some version of a CLTS campaign. In two of those studies, from India and Bangladesh, CLTS activities were combined with subsidies for latrine construction targeted to the poorest members of the community. The intervention in Cambodia also targeted affordability but using a different financial instrument (microcredit). In Indonesia and in another treatment arm in Bangladesh, researchers examined the effect of CLTS activities conducted in isolation, without any financial transfers to the community.

This paper is structured to analyze how interventions that target a common, identifiable *mechanism* underlying the improved sanitation adoption decision (e.g., an informational deficiency or financial affordability) perform across the set of country samples where that intervention was applied, instead of summarizing the effect of each study in isolation. Through this approach, we learn about the relative importance of, say, providing financing vs providing community-level information in determining sanitation outcomes based on the pattern of effects observed across studies.

Our emphasis on harmonizing outcome variables and re-analyzing the microdata from each included study distinguishes our approach from more standard systematic reviews common in the medical literature. Applying this stringent inclusion criterion imposes a cost: it greatly limits the coverage of studies we can include, and this paper therefore does not provide a comprehensive review of all types of sanitation interventions.^1^ But re-analysis of microdata is necessary to properly answer the main research question about the relative effectiveness of financial and non-financial instruments, because financial incentives can take different forms: it can be relayed as a direct price subsidy to lower the cost of purchase, or households can be offered a microfinance contract to defray investment costs. Combining these two forms of interventions to infer the role of financial barriers is further complicated by variation in experimental designs across studies. 3 of the 4 included studies rely on straight treatment-control comparisons, while BenYishay et al. (2017) collects willingness-to-pay (WTP) bids using a Becker–DeGroot–Marschak (BDM) mechanism in each treatment arm. The BDM mechanism complicates identification of the effect of the treatment on behaviors downstream of purchase. We resolve the issue by proposing a novel approach to identifying and estimating these effects, which is of interest independently from the rest of the contributions of our article.^2^

To facilitate comparisons across the different studies, we also harmonize outcome and covariate measures. This allows us to enforce the same sample selection criteria across all studies. If financial subsidies are targeted to only poorer households within the community in study 1 but an information campaign is applied to the whole community in study 2, then re-computing the relative effects of the two interventions on the sub-samples with identical income characteristics makes for a more direct, fairer comparison. The definition and measurement of main outcome variables also vary across studies, and need to be harmonized. Utilizing the micro data we re-define “hygienic” latrines using consistent criteria across studies, and re-analyze effects of interventions, to make them more directly comparable.

We find that – across our sample of studies – interventions that target financial affordability generally have much larger effects on sanitation adoption than CLTS-only interventions that focus on addressing deficiencies in information or community-wide motivation without addressing cost barriers. Furthermore, we find the effects on usage of improved sanitation and the practice of open defecation in interventions incorporating financial incentives to be no worse than in interventions featuring information alone. These results contrast with the traditional CLTS principle of avoiding subsidies, based on the belief that providing financial incentives undermine community-wide motivation to invest in improved sanitation and create a culture of dependency instead of self-sufficiency (Harvey, 2011, Kar and Chambers, 2008).

**Table 1:** Intervention Types .

	*Interventions*				
	CLTS only	CLTS+Subsidy	Micro-credit	Market Link	CLTS+Subsidy+Market Link
Bangladesh	✓	✓		✓	✓
Cambodia			✓		
India		✓			
Indonesia	✓				

*Notes:* This table summarizes the different intervention treatment types available for each country. “Market Link” in Bangladesh refers to an intervention designed to reduce market friction by training village Latrine Supply Agents to connect villagers with providers, provide information on quality, etc. In Bangladesh, “CLTS” refers to the Latrine Promotion Program (LPP), a CLTS-like information and motivation campaign conducted at the neighborhood level.

Recent sanitation interventions aimed at increasing coverage have increasingly targeted women, directly or indirectly, in their promotional efforts (Augsburg et al., 2023, Pakhtigian and Pattanayak, 2024, Stopnitzky, 2017). The gender-focused approach has gained traction as women are expected to benefit more and thus have a stronger preference for improved sanitation at home. Here our analysis of heterogeneity by pre-treatment household characteristics, enabled by using study microdata, paint a mixed picture. We see both negative and positive interaction effects between treatments and the share of women within the household. The pattern of heterogeneous responses we observe across interventions and countries is consistent with the idea that financial benefits are more important for women than information and education, but more research is needed to confirm this conclusion. Notably, we do not see a significant pattern of heterogeneity with respect to household poverty status.

Relative to some of the best-known meta-analyses of CLTS programs, Cameron et al. (2022) and Whittington et al. (2020), we expand the focus to interventions based on different mechanisms by which sanitation adoption decisions are affected, including affordability, supply or market frictions, and product market information failures. While these studies emphasize health outcomes, we investigate adoption and behavior. Relative to Garn et al. (2017), who also consider a wider range of intervention types, we use study microdata, which allows us to harmonize sample selection, outcome, and covariate measurement across study designs. This makes the represented populations comparable on our sample selection criteria and allows us to investigate heterogeneity in the behavioral response to the interventions by household characteristics. We contribute to the recently fast-growing literature on BDM, with notable contributions including Berry, Fischer, and Guiteras (2020), Cole, Fernando, Stein, and Tobacman (2020), and Jack, McDermott, and Sautmann (2022), by showing how to identify and estimate treatment effects for non-purchase outcomes in research designs combining BDM with a randomized evaluation of a treatment or treatments.

The paper proceeds as follows. Section 2 describes the criteria for study inclusion, followed by a description of the intervention types in Section 3. Details of the intervention programs and the experimental design are included in Section 4. Section 5 explains our harmonization strategy for outcomes and sample selection. Section 6 details our approach to estimating the relevant treatment effects, including identification and estimation of treatment effects on non-purchase outcomes in joint RCT-BDM designs. Section 7 presents our main results and examines heterogeneous treatment effects. Section 8 concludes.

## 2 Review methodology

### 2.1 Criteria for the inclusion of studies

We include studies meeting three criteria. First, studies must evaluate an intervention intended to increase the adoption of sanitation products, as opposed to the usage of existing products. Second, the assignment to treatment under the intervention or interventions must be randomized. Lastly, microdata from the studies must be available to allow us to harmonize outcome measures as well as pre-treatment household characteristics. We screen on the availability of the latter so that we can describe how responses to the different intervention types differ across subgroups of the study population.

### 2.2 Search strategy and selection process

To arrive at the set of studies we include in our analysis, we use reference tracking applied to the three aforementioned prominent meta-analyses in the literature on improving sanitation adoption. For each of these meta-analyses, we performed (1) one round of backward reference tracking, looking for studies meeting our inclusion criteria in the meta-analysis’s references, and (2) one round of forward reference tracking, looking for studies meeting our inclusion criteria among studies citing the meta-analysis. Beginning with (Garn et al., 2017), we found 64 studies evaluating sanitation-promotion interventions, including subsidies, supply-side support, education, and demand encouragement, often through Community-Led Total Sanitation (CLTS) programs. Of these 64, 12 were randomized controlled trials, of which three have microdata available, including pre-treatment characteristics: Cameron et al. (2013)’s study carried out in Indonesia, Guiteras et al. (2015) in Bangladesh, and Patil et al. (2014) in India. Forward reference tracking additionally identified (BenYishay et al., 2017) in Cambodia. We followed the same procedure for the (Cameron et al., 2022)^3^ and Whittington et al. (2020) meta-analyses, and although these did result in more randomized evaluations of CLTS programs, none had microdata available.

### 2.3 Overview of contexts

The studies that satisfied our inclusion criteria were conducted in countries and contexts that are extremely important for understanding the spread of improved sanitation globally. The regions represented in our surveys – South Asia and South-East Asia – accounted for over half the world’s population practicing open defecation in 2022 (WHO/UNICEF, 2023). These specific countries combine large populations (almost a quarter of the world’s population among them) with high rural population densities. Because the negative effects of poor sanitation are likely larger in dense populations (Contreras et al., 2022, Hathi et al., 2017), open defecation in the countries studied here represents a particular threat to health. These countries are also important to study because they represent some of the largest gains in improved sanitation in recent years. According to the most recent DHS survey data available, the rate of open defecation in the rural population has fallen in India from 73.8% in 2005–06 to 25.5% in 2019–21, in Indonesia from 32.1% in 2007 to 13.9% in 2017, in Bangladesh from 20.2% in 2007 to 2.5% in 2017–18, and in Cambodia from 77.8% in 2005 to 14.0% in 2021-22 (DHS Program, 2024).^4^

## 3 Types of interventions

Table 1 provides an at-a-glance overview of the different intervention types present in each study included in our meta-analysis by country. In this section, we describe each of the intervention types in detail.

### 3.1 Community-Led Total Sanitation (CLTS)

Community-Led Total Sanitation (CLTS) is described by two of its early pioneers as an approach to ending open defecation, which emphasizes collective rather than individual behavior change and a participatory approach that engages community members to take ownership of their sanitation and hygiene behavior change (Kar & Chambers, 2008). The standard approach to CLTS, as described in Kar and Chambers (2008), involves three stages:

1. Pre-triggering: preparatory steps including community selection, gaining entry to and building rapport with the village;
2. Triggering: activities intended to confront the community with the extent and consequences of open defecation; one core activity is the “transect walk”, in which community members map the location of open defecation sites and human feces;
3. Post-triggering: facilitating the community’s process of deciding how to address the problem of open defecation.

USAID WASHPaLS (2018) describes the core principles of CLTS as

“1. The avoidance of monetary or in-kind hardware subsidies to households 2. The avoidance of prescriptions of any particular latrine hardware designs or models; and 3. The employment of such emotional drivers such as dignity, pride, disgust, or shame to trigger behavior change”. (pg. 6)

CLTS has achieved widespread adoption in the sanitation sector, as evidenced by its inclusion in the rural sanitation policy of 30 countries (USAID WASHPaLS, 2018 Fig. 1). A recent review (Zuin, Delaire, Peletz, Cock-Esteb, Khush et al., 2019) cites several reasons for its popularity, including: (1) the belief that CLTS would yield results quickly without large effort or institutional reform; (2) the perception that CLTS was inexpensive, at least from the point of view of the implementer or government, since the costs were borne by the communities; (3) concordance with a prevailing philosophy of decentralized service provision and community empowerment. Zuin et al. (2019) note specifically that “widespread implementation of CLTS occurred despite mixed evidence on its effectiveness across different contexts, and with limited reliance on robust evidence”.

Venkataramanan, Crocker, Karon, and Bartram (2018) review the literature on the effectiveness of CLTS. They find some evidence of effectiveness in increasing basic latrine coverage and reducing open defecation but less on health benefits and whether effects on coverage can be sustained. They conclude that “the evidence base on CLTS effectiveness ... is weak”. Stuart, Peletz, Albert, Khush, and Delaire (2021) examine which contextual factors are associated with CLTS effectiveness. They find statistically significant associations between accessibility (population density, remoteness of community, access to construction materials) and literacy, but the signs of these interactions differ across countries.

While CLTS or a CLTS-like intervention was implemented in three of the four studies, as seen in Table 1, there were a number of differences across the three countries. Indonesia’s implementation stuck the closest to the abovementioned principles, eschewing subsidies and focusing on reducing open defecation (OD) rather than on the particular type of latrine constructed. In contrast, the CLTS-like intervention conducted in Bangladesh (known as the Latrine Promotion Program (LPP)) emphasized household installation of higher-quality “hygienic” latrines, rather than merely ending OD. One important contextual factor in Bangladesh is the relatively high baseline ownership of and access to latrines (61% and 78%, respectively, of households in control villages). This may have limited the scope of possible effects of a purely demand-side intervention as compared to contexts with very low ownership and access. In India, CLTS was offered combined with financial incentives in the form of subsidies. No “pure” (no-subsidy) CLTS-type intervention was conducted.

### 3.2 Subsidy

Subsidies, at times, offered in conjunction with demand-driven interventions like CLTS, continue to be an important tool for increasing access to improved sanitation facilities, particularly in low-income communities. Important research and policy questions include how effective subsidies are in increasing coverage and reducing open defecation, how to design “smart subsidies” to improve targeting (Andres, Thibert, Lombana Cordoba, Danilenko, Joseph et al., 2019), and how subsidies can interact with traditionally subsidy-free methods such as CLTS (Trimmer et al., 2022).

In our study sample, subsidies were offered in conjunction with CLTS-like interventions in both the Bangladesh and India studies, as seen in Table 1. One important limitation of these studies is that they do not address the potential for a dynamic effect where current subsidies reduce the effectiveness of future non-subsidy interventions. As noted in Venkataramanan et al. (2018), this possibility of dynamic crowding out is a common concern in the CLTS literature.^5^

### 3.3 Market link

The market link intervention was conducted in a randomly selected subset of villages in the Bangladesh study. The intervention was designed to approximate some elements of the “supply-side approach” to sanitation provision that is popular among NGO implementers in certain contexts.^6^ It attempts to reduce a market friction by identifying “Latrine Supply Agents (LSAs)”, connecting them to local masons who are skilled at building latrine parts, training them on parts quality, availability, and prices, and finally placing them in certain randomly chosen villages to relay that information to others, and act as an “expert” point of contact with community members. The intervention design was intended to create a connection between the supply-side providers of sanitation parts and potential consumers so that buyers have more (reliable) information on the quality and availability of components and how to procure them.

The implementation partner typically hired village residents who worked in trades such as masonry, construction, or carpentry and trained them as LSAs. LSAs provided all village residents (regardless of whether they were in the subsidy treatment arm) with information on where to purchase a quality latrine, how to assess the quality of a latrine, how to install a purchased latrine, and how to maintain and repair an installed latrine. Given LSA’s presence in the village, it is likely that they served as points of contact for all sanitation-related questions and likely encouraged improved sanitation behavior more generally.

### 3.4 Micro-credit

In Cambodia, households in treatment villages were offered a 12-month loan for the purchase of a set of latrine components. Labor and installation were not included, nor was a superstructure. Households in control villages were offered the same product, but with payment to be made on delivery. In both types of villages, the household’s maximum willingness to pay (WTP) was elicited through a BDM mechanism. In BDM, the household makes a bid b. This bid is then compared against a random price draw d. If d>b, the household cannot purchase the item. If d≤b, the household purchases the item at the random draw price d. In principle, BDM is incentive-compatible: it is in the household’s best interests to reveal their true maximum WTP, i.e., to bid b=max WTP. In the analysis, WTPs are made comparable across treatment arms by deflating the payment stream by the cost of funds for the MFI providing the loans. Because the household’s actual purchase depends on the random price draw, we construct a “synthetic purchase” variable at the implementer’s break-even price of USD 40. All households bidding the equivalent of USD 40 or more are coded as having purchased since they would have purchased at a fixed offer price of USD 40.

## 4 Intervention details and experimental design

In this section, we describe how the intervention types discussed in the previous section were implemented in each study, and the experimental design. Table 2 provides additional specific details, including precise geographic location, unit of random assignment, stratification in the experimental design, compliance rates in the treatment group and contamination rates in the control group, average duration of the treatment, survey timing, number of villages, and attrition rates by treatment status.

**Table 2:** Interventions .

	*Country*			
	Bangladesh	Cambodia	India	Indonesia
*A. Intervention and Experiment Design*				
Location	1 rural subdistrict in Rajshahi	Kampong Thom province	2 rural districts in Madhya Pradesh	8 rural districts in East Java
Unit of assignment	Village, Neighborhood and Household	Village	Village	Village
Stratification	Union	No	Block	Sub-district
Treatment group compliance	100%	100%	100%	66%
Control group contamination	0%	0%	25%	14%
Average exposure period	4 months	N/A	6 months	24 months

*B. Sample and Timeline*				
Baseline survey	Jan-Mar 2012	Jan-Apr 2013	May–July 2009	Aug–Sept 2008
Endline survey	May-Aug 2013	Dec 2014	Feb–April 2011	Nov 2010–Jan 2011
Number of villages	107	30	80	160
Treatment attrition rate	N/R	N/R	7.9%	4.4%
Control attrition rate	N/R	N/R	7.4%	4.1%

*Notes:* This table summarizes the intervention design, experimental design, and data for all four studies. Panel A presents the geographic location and describes the experimental design. Panel B presents the timeline for the data collection, sample size, and attrition levels for each country. “N/A” means not applicable; “N/R” means not reported. Details on intervention and experiment design from individual countries can be found in Guiteras et al. (2015) for Bangladesh; BenYishay et al. (2017) for Cambodia; Patil et al. (2014) for India, and for Indonesia (Cameron, Olivia, & Shah, 2019). Cambodia: the treatment was a one-time event (marketing and sales exercise), so the average exposure period does not apply.

### 4.1 Guiteras et al. (2015), Bangladesh

#### **Program**.

The Bangladesh interventions were conducted among all households in four rural unions (4th-level administrative division) in Tanore upazila (sub-district, 3rd-level administrative division), Bangladesh. An important difference in context from the other studies is that baseline ownership of and access to basic latrines was relatively high: 61% of households in control villages owned a latrine at baseline, and 78% had access to a latrine. As a result, the intervention in this study emphasized ownership of and access to sanitation in terms of higher-quality “hygienic” latrines, as compared to the emphasis on ending open defecation in traditional CLTS. Our harmonization strategy extends this definition to the other studies (see Section 5).

There were three basic types of intervention, which were offered separately or in combination according to the treatment arm a household was assigned to:

• Latrine-promotion program (LPP): a CLTS-like information and motivation campaign conducted at the neighborhood level
• Subsidy: household-level lottery for a ≈ 75% discount on latrine components
• Market link: Train villager as a “Latrine Supply Agent” to provide neighbors with information to neighborhood residents on latrine availability and quality at local suppliers and how to install and maintain a latrine. See Section 3.3 for the full details.

These interventions were implemented in partnership with the Village Education Resource Centre (VERC), an NGO with a long history of involvement in sanitation in Bangladesh. All households could participate in LPP and the market link intervention, but the least-poor quartile of households (based on landholdings) were not eligible for subsidies.

#### **Experimental design**.

The design for this study was somewhat complex; we provide a brief summary here and a diagram in Appendix Figure A5. There were three tiers of randomization at the village, neighborhood, and household levels. At the highest level, villages were assigned to broad treatment categories: Control, LPP Only, LPP + Subsidy, LPP + Subsidy + Market Link, and Market Link Only. At the second level, within Subsidy villages, subsidy saturation (the share of eligible households winning a discount voucher) was randomized at three levels: Low (approximately 25% of eligible households would win a subsidy voucher); Medium (approximately 50%); High (approximately 75%). Households in Subsidy villages participated in a public lottery for a subsidy voucher, with the share of winners given by the neighborhood’s saturation level. A second, independent public lottery provided free corrugated iron sheets for building the latrine’s walls and roof (colloquially described as “tin”). This was conducted in all Subsidy villages, with a constant (i.e., independent of the neighborhood’s saturation level) win probability of 50%. The 2 × 2 household level lotteries created 4 price points for eligible households in Subsidy villages (won both latrine voucher and tin, won latrine voucher only, won tin only, won neither).

### 4.2 BenYishay et al. (2017), Cambodia

#### **Program**.

The Cambodia intervention was conducted in 30 villages in Kampong Thom province, Cambodia. The study population consisted of households who did not own a latrine at baseline. For harmonization purposes, we enforce this restriction across all studies (see Section 5). Households in treatment villages were offered a microfinance loan of 12 monthly payments to finance the purchase of a set of latrine components. Households in control villages were required to pay the full price on delivery. Latrine marketing and sales were implemented by iDE Cambodia, an NGO, while the loan was implemented by VisionFund Cambodia, an MFI.

#### **Experimental design**.

As described in Section 3.4, villages were randomized into treatment (micro-credit loan) and control (cash-on-delivery). All study households participated in the BDM exercise to elicit willingness to pay for a set of latrine components.

### 4.3 Patil et al. (2014), India

#### **Program**.

India’s Total Sanitation Campaign (TSC) was a large-scale nationwide program initiated by the Government of India (GoI) in 1999 as a restructuring of the Central Rural Sanitation Program (CRSP). The Ministry of Rural Development and the central government implemented TSC to “improve the general quality of life in rural areas and accelerate sanitation coverage in rural areas” (UNICEF and CBGA, 2011).

The program focused on information and education to increase household demand for sanitation. Additionally, school sanitation and hygiene education (SSHE) was emphasized as a starting point to encourage a wider acceptance of sanitation and hygiene practices. The program also recognized the importance of local leadership and integrated rewards to encourage participation through the Nirmal Gram Puraskar (NGP) in October 2003. The NGP awards are given to districts, blocks, and GPs (Gram Panchayats) that have achieved 100 percent sanitation coverage of individual households, 100 percent school sanitation coverage, are free from OD and conduct clean environment maintenance.

The TSC remained the Indian government’s flagship policy for over a decade. During this time period, when the study was conducted, the TSC projects were scaled significantly, and by 2012, the program was operational in 572 rural districts. Although the TSC made some progress, it suffered from being a relatively low priority and ineffective resource deployment. In 2012, the program was replaced by the Nirmal Bharat Abhiyan, which was relaunched in 2014 as the Swachh Bharat Abhiyan.

#### **Experimental design**.

The experiment was implemented in the Indian state of Madhya Pradesh to measure the impact of the TSC implemented with capacity-building support, which included subsidies to households. The study design was a cluster randomized controlled trial with randomization at the village level. The study population included a total of 80 villages selected from 19 Blocks spread across two rural districts in Madhya Pradesh. Like (Cameron et al., 2022), we combine data across these two districts in our analysis. The program measured sanitation access and ownership outcomes and covariates at the household level before and after the intervention in the two survey waves. The original sample consists of 1,954 households with at least one child under 24 months of age. For additional details on the experiment design, see Patil et al. (2014).

### 4.4 Cameron et al. (2019), Indonesia

#### **Program**.

In Indonesia, the evaluated program is known as Total Sanitation and Sanitation Marketing (TSSM) (Cameron et al., 2013). The TSSM program aims to improve sanitation practices in the rural communities of East Java by generating sanitation demand at scale. The approach significantly differed from previous government sanitation policies of providing infrastructure and/or subsidies, instead focusing on existing sanitation practices and the consequences and implications of such practices, thus generating demand for better sanitation services that the market can then respond to. The TSSM approach consisted of three main components: Community-Led Total Sanitation (CLTS), Social Marketing of Sanitation (SMS), and Strengthening the Enabling Environment. Cameron et al. (2019) specifically evaluate the CLTS component, which is captured in their microdata.

#### **Experimental design**.

The TSSM program in Indonesia was implemented in rural East Java. Eight of 29 rural districts in East Java participated in the evaluation, with a total of 160 villages participating. The Indonesian evaluation utilized a randomized design but was unusual in that the program was evaluated when implemented at scale across the province of rural East Java. As outlined by Cameron et al. (2013), within each participating district, the project team randomly selected ten pairs of villages. Each pair comprised one treatment village and one control village from the same sub-district. The original sample consists of approximately 2000 households across 160 rural villages. For additional details on the sampling strategy and data collection, see Cameron et al., 2019, Cameron and Shah, 2010 and Cameron et al. (2013).

## 5 Harmonization

In this section, we describe our harmonization procedures for measuring outcomes and selecting samples to facilitate comparison across studies.

### 5.1 Outcomes

To analyze the treatment impact of each intervention type (described in Section 3) on individual households, we consider four main outcomes that capture the sanitation ownership status and changes in the household’s sanitation behavior and practices.

#### **Sanitation ownership.**.

Our key outcome of interest is sanitation uptake, which we capture using a measure of sanitation ownership for the household. Specifically, whether a household has a sanitation facility inside or within the house compound or property. In each of the four study contexts, we define the household status of sanitation ownership using an indicator that takes value one if the survey respondent indicates the primary (or main) sanitation facility used by household members at the time the survey is conducted is located either within the house or outside the home but on the homestead property and takes the value zero otherwise. In addition to the location, for comparability with (Guiteras et al., 2015)’s focus on hygienic latrine access, we code as zero responses that indicate primary sanitation to be an open field (when relevant) and/or hanging latrines, buckets, and mound latrines.

Our sanitation ownership variable can be thought of as synonymous with the household’s general improved sanitation access, with an additional restriction on the facility’s location. However, we note that in many contexts, a household may not necessarily own the facility, even if it is located on the property. For example, this may be the case if a household rents the house in which it resides. In addition, our outcome variable incorporates information on households owning suitable sanitation infrastructure. Appendix B describes how we construct the sanitation ownership outcome variable in each context.

#### **Sanitation behavior.**.

In addition to the sanitation ownership of the household, we also measure the sanitation practices of household members as captured by sanitation usage and open defecation. For *‘Usage’*, we construct an indicator variable that takes value one if the survey respondent indicates the use of a sanitation facility by adult men and adult women separately within the household. To capture usage behavior accurately, in our construction, we do not distinguish between the facility’s location, i.e., the household members’ usage is not restricted to their own facility. In this way, we allow households to use their neighbors’ toilets and/or public facilities. In three of the four studies (except Cambodia), we can distinguish the sanitation usage behavior of adults by gender. We construct separate variables to capture differences in sanitation usage by gender in response to treatment exposure.

In addition to usage behavior, we measure open defecation (OD) practices by all the household members, adults, and children. The variable *‘Any OD’* is an indicator variable that takes value one if the survey respondent indicates that any member within the household practices OD either always or occasionally and is zero otherwise.^7^ Further details on the construction of sanitation usage and OD practice for each of the study contexts can be found in Appendix B.

### 5.2 Data and sample selection

The data used in our analysis were generated from field experiments in all four countries. Table 2 presents the random assignment and data collection details. For comparability with (BenYishay et al., 2017), we restrict the samples from all studies to households that did not own a latrine at baseline. This also matches specifications that feature prominently in Cameron et al. (2022), e.g., Table 2 in that paper.

In Bangladesh, beginning with the universe of 19,882 households in the study communities who were successfully surveyed, we first restrict to the 13,708 households eligible for subsidies, excluding ineligible households in the top 25% of landholdings. We further restrict to the random 50% subsample of households selected for the full baseline survey, for which we have all covariates, leaving us with 6,244 households. Finally, in our analysis, we restrict to the 4,628 of these households who do not own an improved latrine at baseline. 61% of households in control villages owned a latrine at baseline.

The intervention in Cambodia was targeted solely at households without a latrine. Though we do not directly measure village sanitation coverage at baseline, the village-level sample selection was designed to be representative of rural Kampong Thom province. The DHS survey taken shortly before (2010) the study baseline reports 37.7% of households in Kampong Thom had an improved sanitation facility and an open defecation rate of 61.2% for the province (DHS Program, 2024). Like (BenYishay et al., 2017), our analysis focuses on the sample of 1,383 households without sanitation at baseline.

In Indonesia, we start with a sample of 1,898 households interviewed in both the baseline and follow-up surveys.^8^ In the baseline sample, 978 households had access to improved sanitation at home, while 919 households did not, amounting to a 51.56% baseline sanitation coverage. In our analysis, we restrict to the sub-sample of households without improved sanitation at baseline, yielding a sample of 917 households and an endline sanitation prevalence of 15.30% in the control group.

In India, our sample comprises 1,654 households interviewed in both the baseline and follow-up surveys.^9^ In the baseline sample, only 221 households reported having sanitation at home, while the majority, 1433 households, lacked such access. In contrast to Indonesia, the baseline sanitation coverage was substantially lower at 13.36%. Like Indonesia, we restrict the sample to households without an improved sanitation facility at baseline, yielding a sample of 1433 households and an endline sanitation prevalence of 6.83% in the control group.

## 6 Empirical methodology

In this section, we describe how we estimate the treatment effects reported in the subsequent sections. This is straightforward given the research designs of Cameron et al., 2019, Guiteras et al., 2015, and Patil et al. (2014). The inclusion of a BDM, with its random purchase price, in BenYishay et al. (2017), however, requires us to develop new theory to identify and estimate the effects of the microfinance treatment on the usage and open defecation outcomes. Section 6.2 provides this theory. We perform our re-analyses separately within each study’s dataset and then present the set of results together, facilitating comparison across intervention types.

### 6.1 Guiteras et al. (2015), Patil et al. (2014), Cameron et al. (2019)

For all studies except BenYishay et al. (2017), we estimate the relevant average treatment effects as the coefficient β in the linear regression specification below,

(1) $$Y_{ijs}=\alpha +\beta T_{ijs}+\gamma_{s}+{ɛ}_{ijs}$$

where $Y_{ijs}$ is an indicator for each of the outcomes of interest for a given household i in village/community j in stratum s in the endline survey wave and $\gamma_{s}$ is a stratum fixed effect.^10^ We use the regression specification in Eq. (1) separately for each study in our re-analysis. Since we expect household outcomes to be correlated within assigned treatment units, all estimates include robust standard errors for each study’s parameter β clustered at the relevant village or community level.

### 6.2 BenYishay et al. (2017)

In Cambodia, the BDM mechanism requires us to develop additional theory to identify and estimate effects for outcomes other than purchase that are compatible with those discussed above. We begin by defining two potential outcome functions. The first is

$$Buy_{i}\left(t,P\right),$$

an indicator for whether household i would buy improved sanitation at price P as a function of treatment status t∈{0,1}. t=1 indicates that improved sanitation can be financed, and t=0 represents the status quo where it must be fully paid for at the time of purchase. The second is

(2) $$Y_{i}\left(buy\right),$$

an indicator for whether household i engages in behavior Y (for instance, open defecation) as a function of an indicator buy specifying whether the household owns improved sanitation (buy=1) or not (buy=0). Importantly, by writing (2), we rule out that price or assignment to the financing treatment has a direct effect on behavior Y. Excluding P from $Y_{i}\left(\cdot \right)$ for all i rules out the possibility that paying, for example, a lower price for improved sanitation would influence Y by loosening the household’s budget constraint.^11^

We would like to evaluate the following types of treatment effects

(3) $$E\left[Y_{i}\left(Buy_{i}\left(1,P\right)\right)-Y_{i}\left(Buy_{i}\left(0,P\right)\right)\right]$$

where, for compatibility with the other studies, we approximate effects on uptake of improved sanitation products at the market price by setting P to implementation partner’s breakeven price of 40 USD (see Section 3.4).

Since $T_{i}$ is assigned independently of all other random variables,

$$E\left[Y_{i}\left(Buy_{i}\left(t,P\right)\right)\right]=E\left[Y_{i}\left(Buy_{i}\left(t,P\right)\right)|T_{i}=t\right]$$

so we can consider the treatment and control groups separately. By the law of iterated expectations

$$E\left[Y_{i}\left(Buy_{i}\left(t,P\right)\right)|T_{i}=t\right]$$

can be decomposed as

$$E\left[Y_{i}\left(1\right)|Buy_{i}\left(t,P\right)=1,T_{i}=t\right]P\left(Buy_{i}\left(t,P\right)=1|T_{i}=t\right)+\;E\left[Y_{i}\left(0\right)|Buy_{i}\left(t,P\right)=0,T_{i}=t\right]P\left(Buy_{i}\left(t,P\right)=0|T_{i}=t\right).$$

$Buy_{i}\left(T_{i},P\right)$ is observed as $WTP_{i}\geq P$ for all i, where $WTP_{i}$ is data obtained from the BDM mechanism, so that

$$P\left(Buy_{i}\left(t,P\right)=buy|T_{i}=t\right)=P\left(WTP_{i}\geq P|T_{i}=t\right),$$

which is identified and directly estimable.

It remains to identify the functions

$$E\left[Y_{i}\left(buy\right)|Buy_{i}\left(t,P\right)=buy,T_{i}=t\right].$$

Under the BDM mechanism the observed decision to buy, $Buy_{i}$, depends on whether the draw price $d_{i}$ is less than or equal to i’s willingness to pay. So

(4) $$Y_{i}=Y_{i}\left(Buy_{i}\right)=Y_{i}\left(1\left\{d_{i}\leq WTP_{i}\right\}\right).$$

For a given level of $WTP_{i}=w$,

$$E\left[Y_{i}|WTP_{i}=w,T_{i}=t,d_{i}\leq w\right]=E\left[Y_{i}\left(1\right)|WTP_{i}=w,T_{i}=t,d_{i}\leq w\right]=E\left[Y_{i}\left(1\right)|WTP_{i}=w,T_{i}=t\right],$$

where the first equality follows from Eq. (4) and the second from independent assignment of $d_{i}$. Similarly

$$E\left[Y_{i}|WTP_{i}=w,T_{i}=t,d_{i}>w\right]=E\left[Y_{i}\left(0\right)|WTP_{i}=w,T_{i}=t\right].$$

To obtain $E\left[Y_{i}\left(buy\right)|Buy_{i}\left(t,P\right)=buy,T_{i}=t\right]$, we need to integrate $E\left[Y_{i}|WTP_{i}=w,T_{i}=t,Buy_{i}=buy\right]$ over the distribution of $WTP_{i}|Buy_{i}\left(t,P\right)=buy,T_{i}=t$:

$$\int E\left[Y_{i}|WTP_{i}=w,T_{i}=t,Buy_{i}=buy\right]dF\left(w|Buy_{i}\left(t,P\right)=buy,T_{i}=t\right).$$

It will be convenient to express this as

(5) $$\int E\left[Y_{i}|WTP_{i}=w,T_{i}=t,Buy_{i}=buy\right]\Psi \left(w,buy,t\right)dF\left(w|Buy_{i}\left(t,P\right)=buy,T_{i}=t,Buy_{i}=buy\right)$$

where

$$\Psi \left(w,buy,t\right)=\frac{dF\left(w|Buy_{i}\left(t,P\right)=buy,T_{i}=t\right)}{dF\left(w|Buy_{i}\left(t,P\right)=buy,T_{i}=t,Buy_{i}=buy\right)}.$$

Eq. (5) is a Ψ(w,buy,t)-weighted version of the expression for $E\left[Y_{i}|Buy_{i}\left(t,P\right)=buy,T_{i}=t,Buy_{i}=buy\right]$. This latter expression is the observed expected value of the outcome of interest for households who *would* buy at P (known from the BDM mechanism), have treatment status t, and are observed purchasing because their willingness to pay was below their draw price. The weight corrects for the fact that households for whom $WTP_{i}$ exceeds the draw price will tend to have higher willingness to pay than the full set of households willing to purchase at P. This is because high-WTP households will also purchase at draw prices substantially higher than P.

Using Bayes rule, we can re-write Ψ(w,buy,t) as

(6) $$\frac{P\left(Buy_{i}=buy|Buy_{i}\left(t,P\right)=buy,T_{i}=t\right)}{P\left(Buy_{i}=buy|WTP_{i}=w,Buy_{i}\left(t,P\right)=buy,T_{i}=t\right)}.$$

Among individuals assigned treatment t, the numerator is the share of individuals purchasing under the BDM among those with higher willingness to pay than P and the denominator is the corresponding share of those individuals with $WTP_{i}=w$. The numerator is identified and easily estimated. The denominator is known because the probabilities of different draw prices are set in the BDM mechanism design, potentially depending on the treatment arm. So for a given level of willingness to pay and treatment status we can calculate the denominator as the probability of drawing a price lower than willingness to pay.

In BenYishay et al. (2017) specifically, the draw prices are 80000, 120000, 160000, and 200000 KHR. In the control group, the probability of drawing each price is 0.25, while in the treatment group, the probabilities are 0.05, 0.45, 0.25, and 0.25, respectively. Note that our inference is conditional on having a willingness to pay greater than or equal to the lowest draw price, 80000 KHR. This is because we never see people with WTP < 80000 purchasing, so $Y_{i}\left(Buy_{i}=1\right)$ will never be observed. This group makes up only 15% of the total Cambodia sample.^12^

Summing up, we write Eq. (3) as

(7) $$E\left[Y_{i}\left(1\right)|Buy_{i}\left(1,P\right)=1,T_{i}=1\right]P\left(Buy_{i}\left(1,P\right)=1|T_{i}=1\right)+E\left[Y_{i}\left(0\right)|\;Buy_{i}\left(1,P\right)=0,T_{i}=1\right]P\left(Buy_{i}\left(1,P\right)=0|T_{i}=1\right)\;-E\left[Y_{i}\left(1\right)|Buy_{i}\left(0,P\right)=1,T_{i}=0\right]P\left(Buy_{i}\left(0,P\right)=1|T_{i}=0\right)\;-E\left[Y_{i}\left(0\right)|Buy_{i}\left(0,P\right)=0,T_{i}=0\right]P\left(Buy_{i}\left(0,P\right)=0|T_{i}=0\right)$$

where the $E\left[Y_{i}\left(buy\right)|Buy_{i}\left(t,P\right)=buy,T_{i}=t\right]$ terms are computed using the weighting procedure described above. All other components use sample counterparts for estimation. We compute standard errors using the bootstrap.

## 7 Results

We begin our assessment by examining the impact of various interventions, as described in Section 3, on sanitation ownership, usage, and OD practices at the household level. In Table 3, each row panel represents a unique intervention identified in our study design. When relevant, we break down the panel to allow for comparisons across different countries or treatment arms. The estimation results within each sub-panel are derived from separate within-country regressions, using Eq. (1) or Eq. (7) to measure the outcomes specified in each column.

Column 1 of Table 3 reports the treatment impact estimates for households owning improved sanitation facilities. It is important to note that owning such facilities does not guarantee changes in behavior. Therefore, we also explore the behavior of adult men and women regarding sanitation usage and the prevalence of open defecation within households in columns 2–4.

**Table 3:** Impact of Treatment on Sanitation Ownership and Behavior .

			Sanitation			Any OD
Intervention	Country		Ownership	Usage (Men)	Usage (Women)	
			(1)	(2)	(3)	(4)
*CLTS*	*Bangladesh*	*Treatment*	0.012	0.150***	0.127***	−0.090**
			(0.014)	(0.051)	(0.046)	(0.045)
		*Sample Size*	1,398	1,273	1,395	1,399
		*Control Mean*	0.055	0.513	0.570	0.644
	*Indonesia*	*Treatment*	0.008	0.026	0.022	−0.028
			(0.023)	(0.029)	(0.029)	(0.028)
		*Sample Size*	911	915	915	915
		*Control Mean*	0.153	0.218	0.244	0.826

*CLTS+Subsidy*	*Bangladesh*	*Treatment*	0.093***	0.192***	0.178***	−0.177***
			(0.016)	(0.046)	(0.038)	(0.038)
		*Sample Size*	2,053	1,881	2,048	2,054
		*Control Mean*	0.055	0.513	0.570	0.644
	*India*	*Treatment*	0.113***	0.090***	0.095***	−0.048***
			(0.024)	(0.023)	(0.024)	(0.016)
		*Sample Size*	1,433	1,433	1,433	1,433
		*Control Mean*	0.068	0.060	0.070	0.969

*Micro-credit*	*Cambodia*${}^{‡}$	*Treatment*	0.338***	−0.005		0.040
			(0.053)	(0.049)		(0.049)
		*Sample Size*	1,383	1383		1383
		*Control Mean*	0.279	0.196		0.822

*Market Link*	*Bangladesh*	*Treatment*	0.008	0.172**	0.213***	−0.147***
			(0.013)	(0.069)	(0.064)	(0.044)
		*Sample Size*	1,182	1,084	1,184	1,188
		*Control Mean*	0.055	0.513	0.570	0.644

*CLTS+Subsidy+Market Link*	*Bangladesh*	*Treatment*	0.101***	0.165***	0.167***	−0.153***
			(0.014)	(0.047)	(0.039)	(0.037)
		*Sample Size*	2,131	1,952	2,123	2,131
		*Control Mean*	0.055	0.513	0.570	0.644

*Notes:* This table presents coefficients for the treatment impact on household-level outcomes among households without a private sanitation facility at baseline. Each panel corresponds to a different intervention type subdivided by each country in our study. Each treatment effect comes from a separate estimation of Eq. (1) for the outcome specified at the start of each column. Household-level outcomes include ownership of an improved sanitation facility (column 1), usage of sanitation by adult household members distinguishing men (column 2) and women (column 3), and whether any household member practices OD (column 4). All specifications in columns 1–4 include stratum fixed effects. ‡ In contrast to the estimates for other interventions, the estimate in column 1 for micro-credit denotes the treatment effect on ownership of the *component parts* to construct a latrine, not including its external superstructure. Following the intervention (BenYishay et al., 2017) found that households in treatment, as well as control, preferred to save for an expensive concrete infrastructure before installing. The results in columns 2 - 4 for micro-credit treatment follow the procedure outlined in Section 6. Robust standard errors, clustered at the randomization level (village/community), are presented in parentheses below each treatment effect coefficient. ∗∗∗p<0.01,∗∗p<0.05,∗p<0.1.

### 7.1 Impact on households

#### Sanitation ownership

In the first column of Table 3, we present results for how the treatment impacts household sanitation ownership. The estimates suggest an overall improvement in sanitation ownership in at least one intervention in each of our studies. When we compare the different interventions, we observe statistically significant positive effects for interventions that offer households a financial benefit, either in the form of a micro-credit loan in Cambodia or a subsidy in the case of India and Bangladesh. The two cases where CLTS was implemented without financial incentives, in Bangladesh and Indonesia, do not significantly increase ownership. Additionally, in Bangladesh, the Market Link intervention alone, which does not feature financial incentives, similarly does not increase ownership by a statistically significant amount. Comparing across all CLTS-type interventions, we find statistically significant positive effects for interventions that combine CLTS with subsidies, which include CLTS + Subsidy and CLTS + Subsidy + Market Link interventions.

In the case of the CLTS+Subsidy intervention type, we find similar positive impacts in India and Bangladesh, where sanitation ownership increased by 11 ppts and 9.3 ppts, respectively. Measured in terms of growth relative to the control group ownership rate, the results are again similar, with ownership increasing by 169% in Bangladesh and 166% in India. In Cambodia, there is also a notable and statistically significant increase in sanitation ownership of 34 percentage points, where the treatment entailed a financial benefit in the form of micro-credit loans. Here, the treatment group sanitation ownership rate is more than double that of the control group, with the ownership rate increasing by 121%.

#### Sanitation usage by adults

Looking at behavior change, we see significant improvements in terms of sanitation usage by both adult men (column 2) and women (column 3) across most intervention types. The effects are substantial when positive and statistically significant, ranging between 10 and 20 percentage point increases in usage rates, which are between and 20 and 135% higher than control group rates. Adding financial incentives does not dampen the overall positive effects of CLTS on usage, as one would expect if financial incentives crowd out intrinsic motivation. In Bangladesh, adding subsidies to CLTS does not result in a statistically different effect on usage and, similarly, adding CLTS and subsidies to the Market Link intervention does not change the effect on usage in a statistically significant way. Indonesia’s CLTS and Cambodia’s microcredit intervention do not impact usage significantly, while India’s combination of CLTS and subsidies does increase usage substantially.

Increases in usage may arise for two reasons. First, there is the direct impact of the interventions on the sanitation usage behavior of adults, as in the Bangladesh CLTS-only group, where the treatment entailed information on the importance of sanitation and we see a significant increase in male and female usage. This result suggests a pure behavior change, where adults increase their use of the existing sanitation facilities. Second, increased usage may result from increased sanitation ownership, as in the CLTS+Subsidy interventions. In some contexts, lack of availability of sanitation may have been a factor limiting adult usage of sanitation. In Bangladesh, specifically, part of the effect of subsidies on usage may be due to individuals using newly-purchased improved sanitation facilities, in which case the effect of CLTS on usage conditional on ownership may be lower when combined with subsidies, while the unconditional effect on usage adding subsidies is slightly higher. From our results, we cannot, in general, disentangle the relative importance of the two mechanisms. Nevertheless, our estimates suggest both factors may be relevant.

We note here that in contrast to the estimates for other interventions, the estimate in column 1 for micro-credit denotes the treatment effect on ownership of the component parts to construct a latrine, not including its external superstructure. Following the intervention, BenYishay et al. (2017) found that households in treatment as well as control preferred to save for an expensive concrete infrastructure before installing, which may explain the lack of effect on usage.

Across all samples, female sanitation usage is higher than that of males. The difference in the control means across columns (2) and (3) indicate systematic gender differences in sanitation usage. This suggests that policy may also impact behavior in a manner that is gender specific. We see relatively weak evidence of this in interventions involving CLTS in Bangladesh, with pure CLTS and CLTS + Subsidy showing slightly higher increases in usage for men, and CLTS + Subsidy + Market Link dampening the usage response for women relative to Market Link alone.^13^

#### Prevalence of open defecation within the household

Lastly, we look at treatment impact on the household’s open defecation (OD) rate. This measure includes the practice of OD by any household member, including children. Although correlated, “Any OD”, differently from the sanitation usage, is perhaps more informative of pure behavior/attitudinal change. This is because, as noted above, increased usage may simply be an outcome of increased access to sanitation through shared facilities.

Here, too, financial incentives do not appear to dampen the decreases in OD generated by non-financial interventions. Adding subsidies to CLTS in Bangladesh nearly doubles the decrease in OD, while adding CLTS and subsidies to the Market Link intervention results in a slightly greater decrease in OD. India’s CLTS+Subsidy intervention also significantly decreases OD. Only microcredit and CLTS in Indonesia do not show a statistically significant decrease in OD.

### 7.2 Subgroup analysis

Having explored heterogeneity in effects by intervention type, we investigate whether there is heterogeneity in response to specific interventions by household characteristics.

Table 4 provides summary statistics for the household characteristics we could harmonize for the majority of our studies. These include literacy of the household head (common to all studies except BenYishay et al., 2017), household size, number or share of women and children in total household size, and poverty of the household. For all studies, the number of children was considered the count of children in the household under the age of 5 at baseline.

For Indonesia, following the description in Cameron et al. (2019), a household was considered poor if it was in the bottom quartile of the study sample for non-landed assets. In India a household is considered poor if its members possess a ration card identifying the household as living below the national poverty line (Cameron et al., 2022). In Bangladesh, following Guiteras et al. (2015) we classify a household as poor if it is landless. In Cambodia, a household is classified as poor if it meets the national (IDPoor) standard for poverty.

In our preferred specification, we modify Eq. (1) to include main effects of household characteristics $X_{ijs}$ as well as interactions with treatment arm indicators:

(8) $$Y_{ijs}=\alpha +\beta T_{ijs}+\eta^{\prime }X_{ijs}+\delta^{\prime }T_{ijs}X_{ijs}+\gamma_{s}+{ɛ}_{ijs}$$

We report and discuss the elements of δ. We make an exception for (BenYishay et al., 2017), where the methodology derived in Section 6.2 does not allow for a simple linear specification as in Eq. (8). Instead, we report the difference in effects for households above the median value of each household characteristic in turn, relative to the effect for households below the median. To avoid conflating the differential treatment effects by number of women and children with different effects by household size, we convert the number of women and children to their respective shares of total household size. Household size and shares of women and children are de-meaned separately for each study, so the level effect of treatment represents the effect at the mean level of that covariate.

**Table 4:** Summary Statistics .

		Country			
Variables		Bangladesh	Cambodia	India	Indonesia
		(1)	(2)	(3)	(4)
*Head is literate*	*Mean*	0.356	-	0.923	0.990
	*Std Dev*	0.479	-	0.267	0.099
	*Sample Size*	3,288	-	855	1,821

*Household size*	*Mean*	3.802	4.394	6.942	4.574
	*Std Dev*	1.298	1.768	2.685	1.277
	*Sample Size*	5,813	1,379	1,654	1,898

*Number of women*	*Mean*	1.844	2.243	3.611	2.341
	*Std Dev*	0.958	1.139	1.708	0.986
	*Sample Size*	6,244	1,383	1,654	1,898

*Number of children*	*Mean*	0.443	0.458	2.654	1.192
	*Std Dev*	0.615	0.612	1.449	0.426
	*Sample Size*	6,244	1,379	1,654	1,898

*Household is poor*	*Mean*	0.463	0.275	0.406	0.244
	*Std Dev*	0.499	0.447	0.491	0.430
	*Sample Size*	6,233	1,383	1,460	1,898

*Notes:* Table displays summary statistics for each study in the analysis shown in Columns 1 through 4. Information on education of the household head was unavailable for Cambodia in Column 2.

**Fig. 1:**
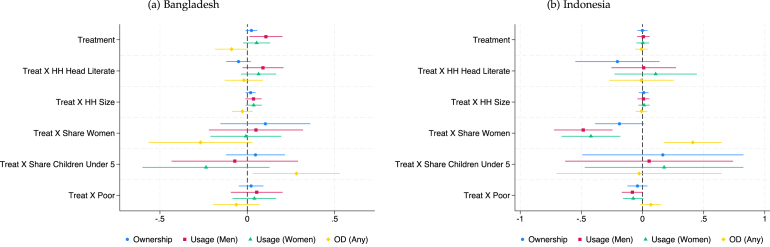
Fully Interacted Specification: CLTS Only. *Notes:* This figure displays estimates of treatment (CLTS) interacted with household-level covariates. Levels and interactions for all covariates are included in the same regression. The outcome variables (ownership, use, and open defecation) are as defined in the text and Appendix B. Continuous covariates (HH size, HH share children under-5 years old, HH share women) are de-meaned (separately for each study), so the level effect of treatment represents the effect at the mean level of that covariate. Results control for fixed effects for geographic units used in stratification and, for Bangladesh, the baseline level of the outcome variable of interest. Standard errors are robust to clustering at the level of randomization (the village).

**Fig. 2:**
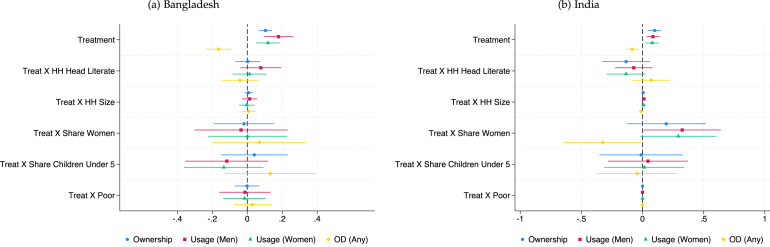
Fully Interacted Specification: CLTS + Subsidy. *Notes:* These figures display estimates of treatment (CLTS + Subsidy) interacted with household-level covariates. Levels and interactions for all covariates are included in the same regression. The outcome variables (ownership, use, and open defecation) are as defined in the text and Appendix B. Continuous covariates (HH size, HH share children under-5 years old, HH share women) are de-meaned (separately for each study), so the level effect of treatment represents the effect at the mean level of that covariate. Results control for fixed effects for geographic units used in stratification and, for Bangladesh, the baseline level of the outcome variable of interest. Standard errors are robust to clustering at the level of randomization (the village).

**Fig. 3:**
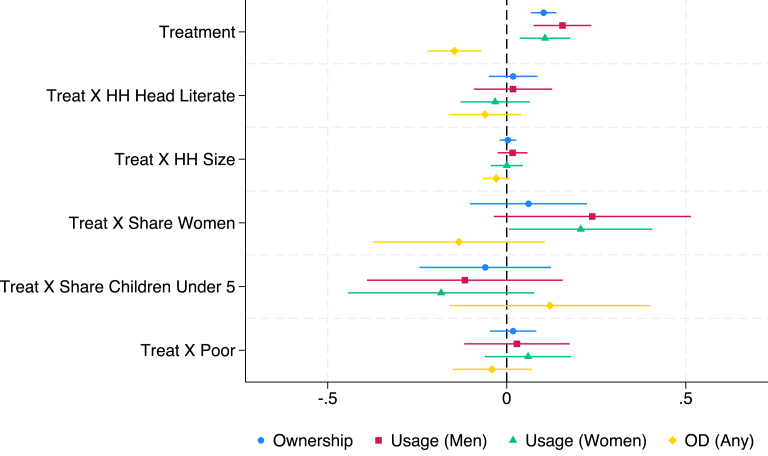
Fully Interacted Specification: CLTS + Subsidy + Market Link Bangladesh. *Notes:* This figure displays estimates of treatment (CLTS + Subsidy + Market Link) interacted with household-level covariates. Levels and interactions for all covariates are included in the same regression. The outcome variables (ownership, use, and open defecation) are as defined in the text and Appendix B. Continuous covariates (HH size, HH share children under-5 years old, HH share women) are de-meaned (separately for each study), so the level effect of treatment represents the effect at the mean level of that covariate. Results control for the baseline level of the outcome variable of interest and fixed effects for geographic units used in stratification. Standard errors are robust to clustering at the level of randomization (the village).

**Fig. 4:**
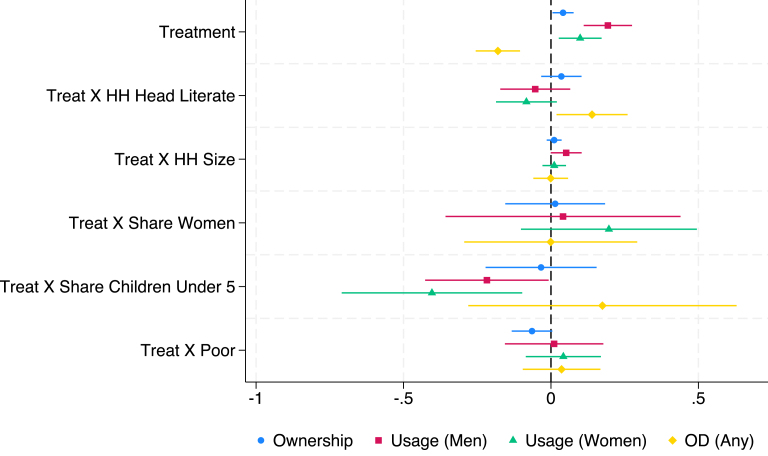
Fully Interacted Specification: Market Link Only Bangladesh. *Notes:* This figure displays estimates of treatment (Market Link) interacted with household-level covariates. Levels and interactions for all covariates are included in the same regression. The outcome variables (ownership, use, and open defecation) are as defined in the text and Appendix B. Continuous covariates (HH size, HH share children under-5 years old, HH share women) are de-meaned (separately for each study), so the level effect of treatment represents the effect at the mean level of that covariate. Results control for fixed effects for geographic units used in stratification and the baseline level of the outcome variable of interest. Standard errors are robust to clustering at the level of randomization (the village).

**Fig. 5:**
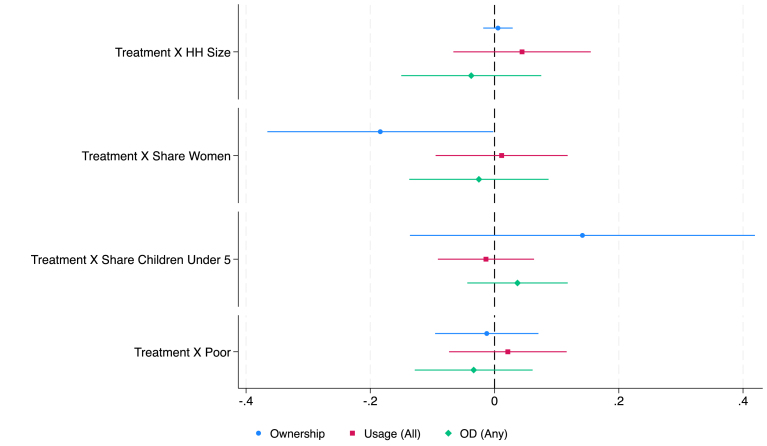
Single Interaction Specification Treatment: Micro-Finance Cambodia. *Notes:* This figure displays estimates of treatment interacted with the household-level covariates indicated, along with 90% confidence intervals. Each interaction is computed in a separate regression. Continuous covariates (HH size, HH share children under-5 years old, HH share women) are de-meaned. The ownership variable is an indicator for whether the household’s willingness to pay was greater than or equal to USD 40 (in net present value for the financing arm). For use and OD, potential outcomes are simulated using the method described in Section 6, with standard errors obtained from bootstrapping with replacement at the village level (500 repetitions). Standard errors are robust to clustering at the level of randomization (the village).

Fig. 1, Fig. 2, Fig. 3, Fig. 4 summarize the results for all countries except Cambodia, with the full regression output presented in tables A1-A4 in Appendix. Fig. 5 summarizes the results for the microfinance intervention in Cambodia, with details in tables A6e, A7e, A8e, and A9e. For comparison with the results in Cambodia, we also provide Appendix Figures A1–A4 and tables A5–A9, which show the outcome of interacting the treatment indicator with one covariate at a time, instead of jointly as we do in Fig. 1, Fig. 2, Fig. 3, Fig. 4.

We highlight some key results in this discussion. In Fig. 1(b), we see that in the Indonesian CLTS intervention, households with a greater share of women and poor households experience significantly smaller treatment effects. However, the opposite is true in India’s CLTS + Subsidy treatment, with households with greater shares of women having larger treatment effects and no heterogeneity by poverty status. The pattern is similar to India’s in Bangladesh’s CLTS and CLTS + Subsidy + Market Link interventions, though heterogeneity effects by share of women are not statistically significant at the 10% level. Across the multiple interventions in Bangladesh, share of children under 5 is typically associated with smaller treatment effects on desired outcomes. Heterogeneity in the microcredit program in Cambodia follows a pattern more similar to Indonesia’s CLTS, with share of women negatively associated with the treatment effect on ownership.

Overall, the message of our heterogeneity analysis is that it would be premature to make targeting recommendations based on the studies included in this re-analysis. The share of women most frequently contributes significant amounts of treatment effect heterogeneity, though whether the effect is positive or negative depends on intervention and country. The results on heterogeneity by share of women, combined with greater control group usage of improved sanitation by women are roughly suggestive of financial benefits being more important for women, as opposed to information and education. However, more research is needed to investigate this conclusion. Other variables overall do not have a significant effect on response to treatment, suggesting the effects for these are more subtle if they are present at all. Again, additional research is necessary to make this conclusion more precise.

## 8 Discussion and conclusion

Given large public health externalities stemming from sanitation-related behavior as identified in the epidemiological literature, interventions to promote improved sanitation have a solid theoretical basis. However, identifying the specific intervention design that would be most effective at improving sanitation coverage in a given context remains elusive, and has been the target of an RCT-based literature which we re-analyze.

One critical distinction between our approach and other systematic reviews and re-analyses is to group intervention types that are common across studies and center the analysis on mechanisms by which each intervention is designed to affect household behavior. Using this approach, we find that interventions like subsidies and microcredit which target financial constraints, generally perform better in promoting adoption of improved sanitation than interventions like CLTS that target information and coordination costs alone. In terms of promoting use of improved sanitation and decreasing open defecation, financial incentives do no worse than their non-financial counterparts, and do not appear to dampen the effects of non-financial incentives. Using within-study heterogeneity analysis we also explore whether targeting sub-populations with specific interventions are likely to enhance effectiveness. We discover patterns of heterogeneity with respect to the share of women in the household, but (surprisingly) not with respect to household poverty status.

Our findings are generally consistent with financial constraints representing an important barrier to improved sanitation adoption. Our results suggest that these constraints may bind more tightly for women, but this pattern is not consistent across all of our studies. Overall, we believe more research applying our approach of harmonizing impact evaluation microdatasets to identify effect heterogeneity by subgroups of households is necessary to arrive at evidence-based targeting recommendations.

Finally, we comment on some implications for research practice. First, we encourage researchers to make data readily available, consistent of course with ethical obligations to protect personally identifying information and to abide by the terms of participant informed consent. There are several free and convenient options to do so (e.g., Dataverse and the Open Science Foundation), among which the WaSH-focused Project W seems particularly promising in this sector (https://aquaya.org/project-w/). Second, researchers can facilitate aggregation of information across studies by anticipating the desire to harmonize datasets when designing surveys and even interventions. Of course, each study will have its own specific needs, but it is often possible to design a survey both to obtain specific pieces of information relevant to that study’s unique questions and to allow for simple harmonization with other studies. For example, a researcher designing a survey could begin with the most recent DHS or MICS water and sanitation module for that country and then add questions as needed for the particular aims of that study.^14^ See Aquaya (2022) and UNICEF and WHO (2018) for recommendations. Finally, we observe that many RCTs compare one intervention type against a control condition. This leads to limited opportunities to compare intervention types within a single context, and the need to extrapolate across trials. However, we recognize that adding contrasts to trials can be challenging in terms of budgets and sample sizes, especially since the social nature of many interventions and the plausibility of demand or health spillovers often require that trials be randomized at a cluster (e.g., village) level. Creative ways of randomizing treatment variants at a finer level, while either avoiding or measuring spillovers, would be a welcome innovation.

## CRediT authorship contribution statement

**Sanghmitra Gautam:** Writing – review & editing, Writing – original draft, Software, Supervision, Project administration, Methodology, Investigation, Formal analysis, Conceptualization. **Michael Gechter:** Writing – review & editing, Writing – original draft, Supervision, Software, Project administration, Methodology, Investigation, Formal analysis, Conceptualization. **Raymond P. Guiteras:** Writing – review & editing, Writing – original draft, Supervision, Software, Project administration, Methodology, Investigation, Formal analysis, Data curation, Conceptualization. **Ahmed Mushfiq Mobarak:** Writing – review & editing, Writing – original draft, Supervision, Project administration, Methodology, Investigation, Data curation, Conceptualization.

## Declaration of competing interest

All authors declare no relevant or material financial interests that relate to the research described in this paper.

## Data Availability

Replication code and data are publicly available on the Harvard Dataverse at https://doi.org/10.7910/DVN/UADUOW .

## Appendix A Supplementary data

MMC S1 Online appendices A and B.

## Footnotes

1 For example, we remain silent about government-led approaches that prioritize large-scale toilet construction, but which have not been evaluated using RCTs. These campaigns work along multiple margins, while our approach examines the specific mechanisms targeted by narrowly designed interventions. Our study also remains silent on smaller-scale policy innovations such as the Haryana government’s 2005 “No toilet, no bride” campaign (Stopnitzky, 2017) or tournaments or prizes like “Open Defecation Free” status that recognizes communities that make progress on sanitation with group-level rewards (Lamba & Spears, 2013).

2 Households with higher willingness to pay are more likely to be observed purchasing at their individual drawn price in a BDM auction than other households who would also purchase at the market price. We therefore developed theory that yields the proper adjustments that must be applied to down-weight purchasers with high WTP and up-weight purchasers with lower WTP, so that the average is representative of the distribution of willingness to pay for households who would purchase at the market price.

3 To be precise, we used Gertler, Shah, Alzua, Cameron, Martinez et al. (2015), which was the pre-publication version of this paper available at the time we conducted our review.

4 We include “hanging latrine” in the open defecation category.

5 We are not aware of RCTs that test this proposition exactly, although Bakhtiar et al. (2023) find that a social influence intervention conducted after the subsidy intervention of Guiteras et al. (2015) had positive effects in the short- and medium-term. See Dupas and Miguel (2017) for a discussion of the potential dynamic effects of subsidies on demand for preventative health goods more generally.

6 In the context of Guiteras et al. (2015), it was not possible to conduct a randomized intervention with suppliers (masons) themselves. First, there was not a sufficient number of masons in the study region for randomization. Second, there were no clear geographic demarcations or well-defined exclusive markets: while masons tended to sell in villages nearer to their own place of business, any mason could, in principle, sell to any village.

7 The extent to which changes in sanitation behavior are captured in *Usage* and *Any OD* outcome variable in Cambodia is limited. In the survey information available, we are unable to document the use (or lack) of shared or community sanitation facilities. If individuals make use of shared and community sanitation in Cambodia, the variable *Usage* would under-report usage behavior, while *Any OD* would over-report the practice of OD by household members.)

8 In Indonesia, randomization was conducted at the village level, stratified by sub-district. Cameron et al. (2019) report partial compliance with the randomization assignment, where 66% of the treatment villages and 14% of control villages triggered exposed to the program. We follow a similar approach to Cameron et al., 2022, Cameron et al., 2019 and estimate the average treatment effect across villages that were assigned to treatment, that is Intention-to-Treat (ITT) estimates.

9 In India, randomization was conducted at the village level stratified by Block. Patil et al. (2014) report full compliance in the treatment group with 100% of treatment villages receiving the program. In contrast, there was some contamination in the control group, where 10 out of the 40 villages, approximately 25%, received the TSC program.

10 The stratum refers to the sub-district in Indonesia, block in India, and union in Bangladesh. The experiment in Cambodia was not stratified.

11 BenYishay et al. (2017) find some evidence of a modest *negative* causal effect of price paid on installation, but not enough to influence our results here noticeably. Similarly, we assume away any peer effects. In other words, we assume that the household’s observed behavior in the actual price regime, where the share of households purchasing in the village depended on the distribution of WTP in the village and the distribution of draws in BDM represents what the household’s behavior would be in a regime with a fixed price of P for all households. The expected value of the BDM draw as implemented was approximately USD 35, so on average, the share of households purchasing at a fixed price of 40 would be slightly lower than the actual share of households purchasing. BenYishay et al. (2017) find a modest *negative* peer effect on installation, so the corresponding positive effect from a lower overall purchase rate could offset the negative price paid effect noted above. As mentioned, for simplicity, we abstract from both.

12 One could also easily estimate bounds on unconditional average treatment effects.

13 The lack of purchase response to the Market Link intervention combined with its substantial effects on usage may initially appear surprising, given the LSA’s focus on provision of information regarding latrines available for households to purchase. However, based on conversations with implementing staff, our understanding is that the LSAs’ status as a “one-stop-shop” for sanitation questions mentioned in Section 3.3 meant that they ended up promoting sanitary practice.

14 In these cases, it is especially useful to provide a survey explication document, describing sources used, how these sources were adapted and modified, and how individual survey questions are aggregated to variables used in the study. As a rudimentary example, see the Followup Survey Explanation document from BenYishay et al. (2017), posted to the Harvard Dataverse at https://doi.org/10.7910/DVN/SQDCJZ.
