# Supplementary material for: To use financial incentives or not? Insights from experiments in encouraging sanitation investments in four countries
Source: World Dev. 2025 Mar;187:106791. doi: 10.1016/j.worlddev.2024.106791 (PMC11659501; doi:10.1016/j.worlddev.2024.106791)
Supplement: MMC S1 — Online appendices A and B. [file mmc1.pdf]

A Appendix Tables and Figures

A.1 Fully Interacted Specification Tables

Table A1: Fully Interacted Specification
Treatment: CLTS

(a) Bangladesh

	(1) Ownership	(2) Usage (Men)	(3) Usage (Women)	(4) Any OD
Treatment	0.024	0.107*	0.054	-0.090
	(0.020)	(0.057)	(0.047)	(0.057)
HH Head Literate	0.022	0.006	0.054	-0.010
	(0.026)	(0.062)	(0.054)	(0.048)
Treatment X HH Head Literate	-0.050	0.090	0.065	-0.019
	(0.042)	(0.071)	(0.061)	(0.066)
HH Size	0.005	-0.032	-0.008	0.024
	(0.009)	(0.021)	(0.022)	(0.019)
Treatment X HH Size	0.019	0.036	0.036	-0.028
	(0.017)	(0.028)	(0.028)	(0.036)
HH Share Women	-0.020	-0.090	-0.099	0.101
	(0.051)	(0.134)	(0.097)	(0.106)
Treatment X Share Women	0.104	0.050	-0.008	-0.267
	(0.155)	(0.162)	(0.122)	(0.179)
HH Share Children Under 5	-0.014	0.050	0.143	0.733***
	(0.081)	(0.107)	(0.108)	(0.112)
Treatment X Share Children Under 5	0.047	-0.072	-0.236	0.281*
	(0.102)	(0.218)	(0.219)	(0.150)
Poor	-0.046	-0.031	-0.039	0.084
	(0.029)	(0.084)	(0.068)	(0.055)
Treatment X Poor	0.022	0.054	0.041	-0.063
	(0.042)	(0.089)	(0.075)	(0.080)
Control group mean	0.062	0.581	0.651	0.633
Num. clusters	107	107	107	107
Num. households	2214	2182	2217	2220

Notes: This table displays estimates of treatment (CLTS + Subsidy) interacted with household-level covariates. Levels and interactions for all covariates are included in the same regression. The outcome variables (ownership, use, and open defecation) are as defined in the text and Appendix B. Continuous covariates (HH size, HH share children under-5 years old, HH share women) are de-meaned (separately for each study), so the level effect of treatment represents the effect at the mean level of that covariate. Results control for the baseline level of the outcome variable of interest and fixed effects for geographic units used in stratification. Standard errors are robust to clustering at the level of randomization (the village). * $p < 0.10$, ** $p < 0.05$, *** $p < 0.01$.

Table A1: Fully Interacted Specification
Treatment: CLTS (Continued)

(b) Indonesia

	(1) Ownership	(2) Usage (Men)	(3) Usage (Women)	(4) Any OD
Treatment	-0.001	0.007	0.002	-0.008
	(0.025)	(0.031)	(0.032)	(0.031)
HH Head Literate	0.144*	0.029	-0.059	-0.029
	(0.077)	(0.088)	(0.150)	(0.087)
Treatment X HH Head Literate	-0.205	0.010	0.108	-0.010
	(0.208)	(0.160)	(0.203)	(0.160)
HH Size	-0.004	-0.008	-0.009	0.003
	(0.017)	(0.023)	(0.022)	(0.022)
Treatment X HH Size	0.010	0.008	0.013	-0.007
	(0.024)	(0.030)	(0.028)	(0.029)
HH Share Women	0.090	0.301***	0.234**	-0.245**
	(0.075)	(0.109)	(0.108)	(0.108)
Treatment X Share Women	-0.188	-0.484***	-0.422***	0.412***
	(0.121)	(0.145)	(0.145)	(0.144)
HH Share Children Under 5	-0.009	0.332	0.268	-0.375
	(0.282)	(0.315)	(0.287)	(0.310)
Treatment X Share Children Under 5	0.166	0.054	0.177	-0.027
	(0.398)	(0.415)	(0.392)	(0.408)
Poor	-0.037	-0.032	-0.059*	0.039
	(0.036)	(0.036)	(0.034)	(0.036)
Treatment X Poor	-0.041	-0.083	-0.076	0.068
	(0.050)	(0.053)	(0.050)	(0.051)
Control group mean	0.156	0.224	0.251	0.251
Num. clusters	150	150	150	150
Num. households	864	868	868	868

Notes: This table displays estimates of treatment (CLTS + Subsidy) interacted with household-level covariates. Levels and interactions for all covariates are included in the same regression. The outcome variables (ownership, use, and open defecation) are as defined in the text and Appendix B. Continuous covariates (HH size, HH share children under-5 years old, HH share women) are de-meaned (separately for each study), so the level effect of treatment represents the effect at the mean level of that covariate. Results control for the baseline level of the outcome variable of interest and fixed effects for geographic units used in stratification. Standard errors are robust to clustering at the level of randomization (the village). * $p < 0.10$, ** $p < 0.05$, *** $p < 0.01$.

Table A2: Fully Interacted Specification
Treatment: CLTS + Subsidy

(a) Bangladesh

	(1) Ownership	(2) Usage (Men)	(3) Usage (Women)	(4) Any OD
Treatment	0.024	0.107*	0.054	-0.090
	(0.020)	(0.057)	(0.047)	(0.057)
HH Head Literate	0.022	0.006	0.054	-0.010
	(0.026)	(0.062)	(0.054)	(0.048)
Treatment X HH Head Literate	-0.050	0.090	0.065	-0.019
	(0.042)	(0.071)	(0.061)	(0.066)
HH Size	0.005	-0.032	-0.008	0.024
	(0.009)	(0.021)	(0.022)	(0.019)
Treatment X HH Size	0.019	0.036	0.036	-0.028
	(0.017)	(0.028)	(0.028)	(0.036)
HH Share Women	-0.020	-0.090	-0.099	0.101
	(0.051)	(0.134)	(0.097)	(0.106)
Treatment X Share Women	0.104	0.050	-0.008	-0.267
	(0.155)	(0.162)	(0.122)	(0.179)
HH Share Children Under 5	-0.014	0.050	0.143	0.733***
	(0.081)	(0.107)	(0.108)	(0.112)
Treatment X Share Children Under 5	0.047	-0.072	-0.236	0.281*
	(0.102)	(0.218)	(0.219)	(0.150)
Poor	-0.046	-0.031	-0.039	0.084
	(0.029)	(0.084)	(0.068)	(0.055)
Treatment X Poor	0.022	0.054	0.041	-0.063
	(0.042)	(0.089)	(0.075)	(0.080)
Control group mean	0.062	0.581	0.651	0.633
Num. clusters	107	107	107	107
Num. households	2214	2182	2217	2220

Notes: This table displays estimates of treatment (CLTS + Subsidy) interacted with household-level covariates. Levels and interactions for all covariates are included in the same regression. The outcome variables (ownership, use, and open defecation) are as defined in the text and Appendix B. Continuous covariates (HH size, HH share children under-5 years old, HH share women) are de-meaned (separately for each study), so the level effect of treatment represents the effect at the mean level of that covariate. Results control for the baseline level of the outcome variable of interest and fixed effects for geographic units used in stratification. Standard errors are robust to clustering at the level of randomization (the village). * $p < 0.10$, ** $p < 0.05$, *** $p < 0.01$.

Table A2: Fully Interacted Specification
Treatment: CLTS + Subsidy (Continued)

(b) India

	(1) Ownership	(2) Usage (Men)	(3) Usage (Women)	(4) Any OD
Treatment	0.099***	0.085***	0.080**	-0.085***
	(0.033)	(0.032)	(0.032)	(0.032)
HH Head Literate	0.071**	0.040	0.054	-0.040
	(0.035)	(0.045)	(0.046)	(0.045)
Treatment X HH Head Literate	-0.134	-0.072	-0.134	0.072
	(0.117)	(0.092)	(0.096)	(0.092)
HH Size	0.006	0.003	0.004	-0.003
	(0.006)	(0.006)	(0.006)	(0.006)
Treatment X HH Size	0.005	0.011	0.009	-0.011
	(0.009)	(0.010)	(0.010)	(0.010)
HH Share Women	-0.049	-0.125	-0.112	0.125
	(0.091)	(0.103)	(0.099)	(0.103)
Treatment X Share Women	0.194	0.324*	0.293	-0.324*
	(0.194)	(0.189)	(0.187)	(0.189)
HH Share Children Under 5	-0.015	-0.044	-0.058	0.044
	(0.107)	(0.099)	(0.100)	(0.099)
Treatment X Share Children Under 5	-0.011	0.045	0.014	-0.045
	(0.205)	(0.195)	(0.196)	(0.195)
Poor	0.000	0.000	0.000	-0.000
	(0.000)	(0.000)	(0.000)	(0.000)
Treatment X Poor	0.001	0.001	0.001	-0.001
	(0.001)	(0.001)	(0.001)	(0.001)
Control group mean	0.106	0.103	0.115	0.897
Num. clusters	79	79	79	79
Num. households	667	667	667	667

Notes: This table displays estimates of treatment (CLTS + Subsidy) interacted with household-level covariates. Levels and interactions for all covariates are included in the same regression. The outcome variables (ownership, use, and open defecation) are as defined in the text and Appendix B. Continuous covariates (HH size, HH share children under-5 years old, HH share women) are de-meaned (separately for each study), so the level effect of treatment represents the effect at the mean level of that covariate. Results control for the baseline level of the outcome variable of interest and fixed effects for geographic units used in stratification. Standard errors are robust to clustering at the level of randomization (the village). * $p < 0.10$, ** $p < 0.05$, *** $p < 0.01$.

Table A3: Fully Interacted Specification
Treatment: CLTS + Subsidy + Market Link
Bangladesh

	(1) Ownership	(2) Usage (Men)	(3) Usage (Women)	(4) Any OD
Treatment	0.103***	0.156***	0.107**	-0.146***
	(0.022)	(0.049)	(0.043)	(0.046)
HH Head Literate	0.022	0.006	0.054	-0.010
	(0.026)	(0.062)	(0.054)	(0.048)
Treatment X HH Head Literate	0.018	0.017	-0.032	-0.061
	(0.041)	(0.066)	(0.058)	(0.061)
HH Size	0.005	-0.032	-0.008	0.024
	(0.009)	(0.021)	(0.022)	(0.019)
Treatment X HH Size	0.003	0.016	0.000	-0.030
	(0.014)	(0.025)	(0.027)	(0.023)
HH Share Women	-0.020	-0.090	-0.099	0.101
	(0.051)	(0.134)	(0.097)	(0.106)
Treatment X Share Women	0.061	0.239	0.207*	-0.134
	(0.099)	(0.166)	(0.120)	(0.145)
HH Share Children Under 5	-0.014	0.050	0.143	0.733***
	(0.081)	(0.107)	(0.108)	(0.112)
Treatment X Share Children Under 5	-0.060	-0.117	-0.183	0.120
	(0.111)	(0.165)	(0.157)	(0.169)
Poor	-0.046	-0.031	-0.039	0.084
	(0.029)	(0.084)	(0.068)	(0.055)
Treatment X Poor	0.018	0.028	0.060	-0.041
	(0.039)	(0.089)	(0.073)	(0.067)
Control group mean	0.062	0.581	0.651	0.633
Num. clusters	107	107	107	107
Num. households	2214	2182	2217	2220

Notes: This table displays estimates of treatment (CLTS + Subsidy + Market Link) interacted with household-level covariates. Levels and interactions for all covariates are included in the same regression. The outcome variables (ownership, use, and open defecation) are as defined in the text and Appendix B. Continuous covariates (HH size, HH share children under-5 years old, HH share women) are de-meaned (separately for each study), so the level effect of treatment represents the effect at the mean level of that covariate. Results control for the baseline level of the outcome variable of interest and fixed effects for geographic units used in stratification. Standard errors are robust to clustering at the level of randomization (the village). * $p < 0.10$, ** $p < 0.05$, *** $p < 0.01$.

Table A4: Fully Interacted Specification
Treatment: Market Link
Bangladesh

	(1) Ownership	(2) Usage (Men)	(3) Usage (Women)	(4) Any OD
Treatment	0.041*	0.193***	0.099**	-0.180***
	(0.022)	(0.049)	(0.044)	(0.045)
HH Head Literate	0.022	0.006	0.054	-0.010
	(0.026)	(0.062)	(0.054)	(0.048)
Treatment X HH Head Literate	0.035	-0.053	-0.083	0.139*
	(0.041)	(0.072)	(0.062)	(0.073)
HH Size	0.005	-0.032	-0.008	0.024
	(0.009)	(0.021)	(0.022)	(0.019)
Treatment X HH Size	0.011	0.052	0.011	-0.001
	(0.015)	(0.032)	(0.024)	(0.035)
HH Share Women	-0.020	-0.090	-0.099	0.101
	(0.051)	(0.134)	(0.097)	(0.106)
Treatment X Share Women	0.014	0.041	0.197	-0.001
	(0.102)	(0.240)	(0.180)	(0.177)
HH Share Children Under 5	-0.014	0.050	0.143	0.733***
	(0.081)	(0.107)	(0.108)	(0.112)
Treatment X Share Children Under 5	-0.034	-0.217*	-0.404**	0.174
	(0.113)	(0.126)	(0.184)	(0.274)
Poor	-0.046	-0.031	-0.039	0.084
	(0.029)	(0.084)	(0.068)	(0.055)
Treatment X Poor	-0.064	0.011	0.042	0.036
	(0.042)	(0.101)	(0.077)	(0.079)
Control group mean	0.062	0.581	0.651	0.633
Num. clusters	107	107	107	107
Num. households	2214	2182	2217	2220

Notes: This table displays estimates of treatment (CLTS + Subsidy) interacted with household-level covariates. Levels and interactions for all covariates are included in the same regression. The outcome variables (ownership, use, and open defecation) are as defined in the text and Appendix B. Continuous covariates (HH size, HH share children under-5 years old, HH share women) are de-meaned (separately for each study), so the level effect of treatment represents the effect at the mean level of that covariate. Results control for the baseline level of the outcome variable of interest and fixed effects for geographic units used in stratification. Standard errors are robust to clustering at the level of randomization (the village). * $p < 0.10$, ** $p < 0.05$, *** $p < 0.01$.

A.2 Marginal Interactions, Figures and Tables

Figure A1: Single Interaction Specification
Treatment: CLTS Only

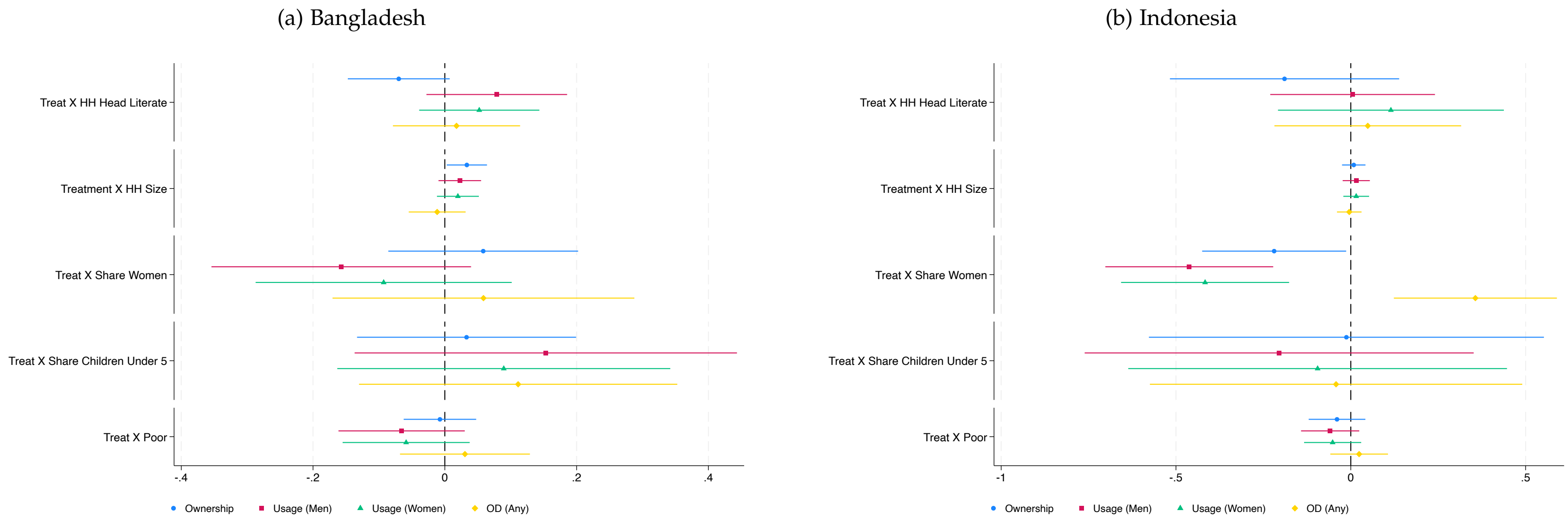

Notes: These figures display estimates of treatment interacted with the household-level covariates indicated, along with 90% confidence intervals. Each interaction is computed in a separate regression. Continuous covariates (HH size, HH share children under-5 years old, HH share women) are de-meaned. Results for Bangladesh control for the baseline level of the outcome variable of interest. Results for Bangladesh and Indonesia include fixed effects for geographic units used in stratification. Standard errors are robust to clustering at the level of randomization (the village).

Figure A2: Single Interaction Specification
Treatment: CLTS + Subsidy

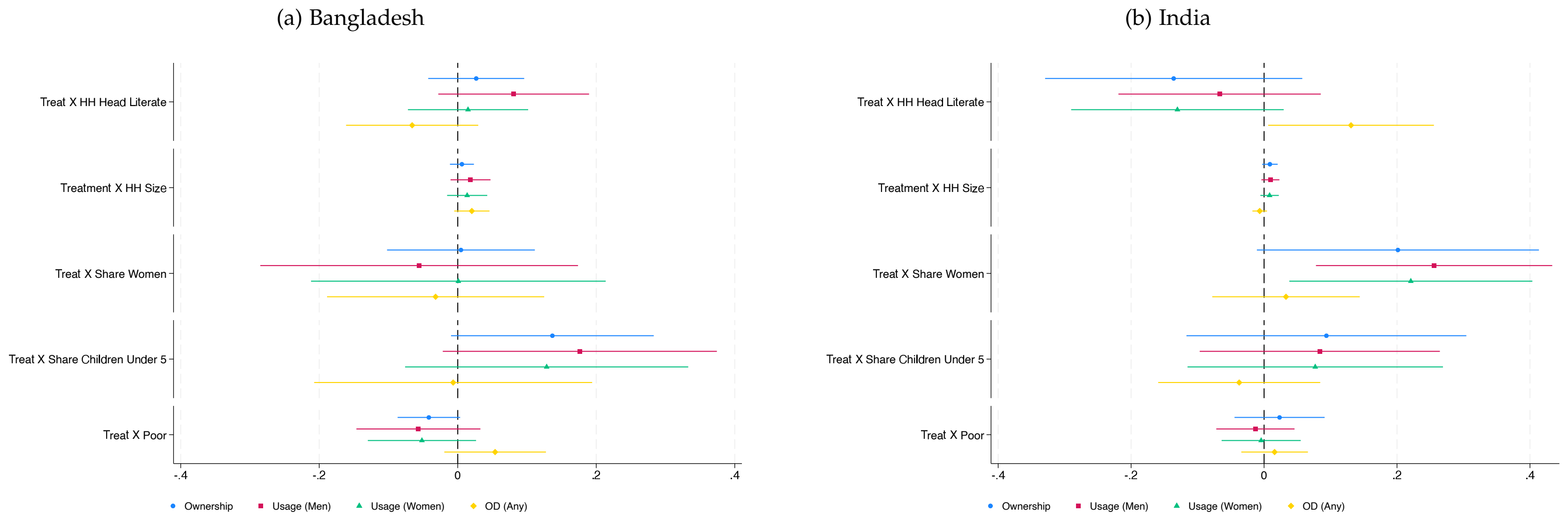

Notes: These figures display estimates of treatment interacted with the household-level covariates indicated, along with 90% confidence intervals. Each interaction is computed in a separate regression. Continuous covariates (HH size, HH share children under-5 years old, HH share women) are de-meaned. Results for Bangladesh control for the baseline level of the outcome variable of interest. Results for Bangladesh and India include fixed effects for geographic units used in stratification. Standard errors are robust to clustering at the level of randomization (the village).

Figure A3: Single Interaction Specification
Treatment: CLTS + Subsidy + Market Link
Bangladesh

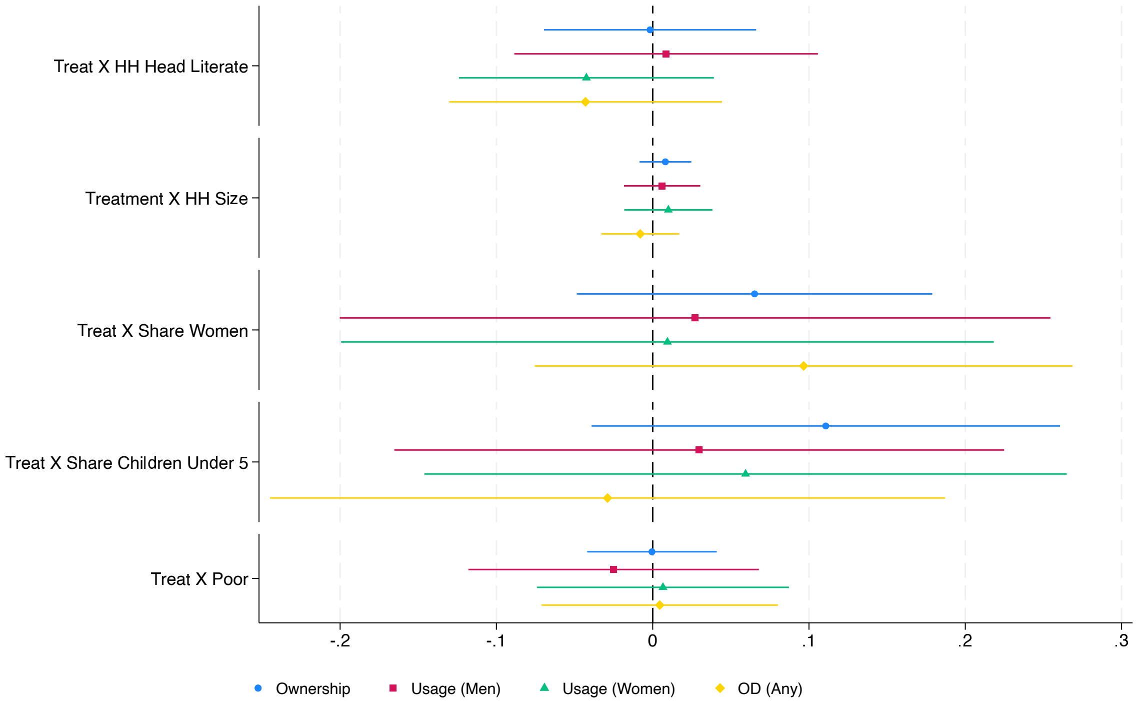

Notes: This figure displays estimates of treatment interacted with the household-level covariates indicated, along with 90% confidence intervals. Each interaction is computed in a separate regression. Continuous covariates (HH size, HH share children under-5 years old, HH share women) are de-meaned. Regressions include fixed effects for the geographic stratification unit and controls for the baseline level of the outcome variable. Standard errors are robust to clustering at the level of randomization (the village).

Figure A4: Single Interaction Specification
Treatment: Market Link Only
Bangladesh

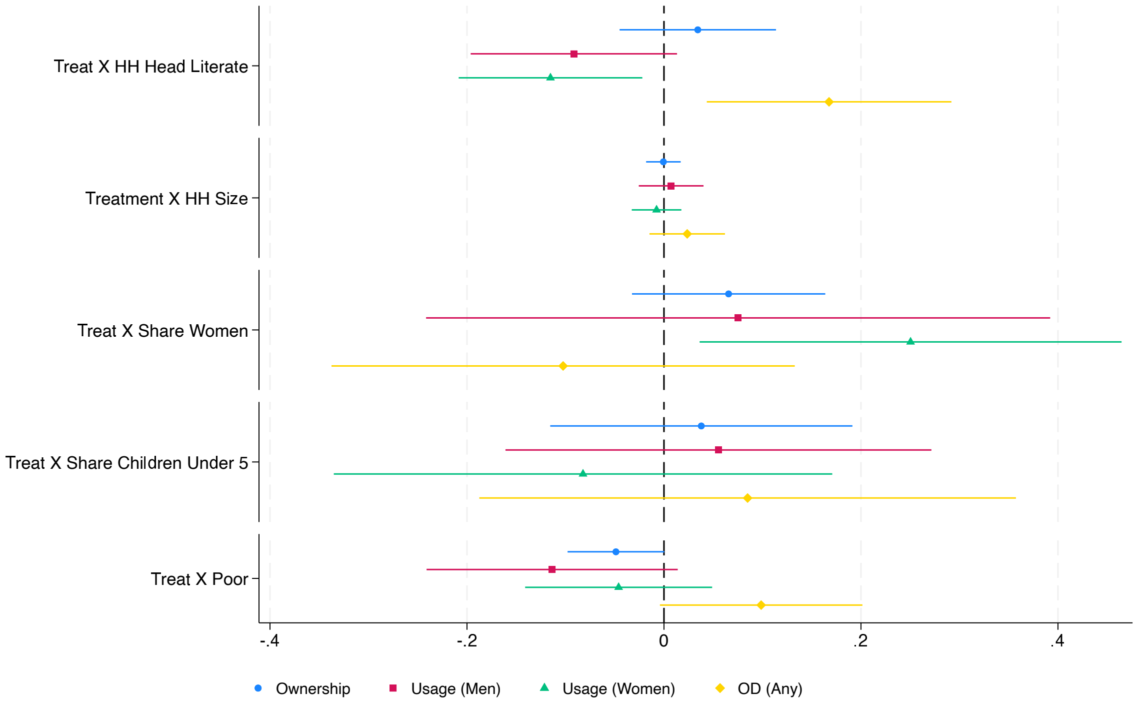

Notes: This figure displays estimates of treatment interacted with the household-level covariates indicated, along with 90% confidence intervals. Each interaction is computed in a separate regression. Continuous covariates (HH size, HH share children under-5 years old, HH share women) are de-meaned. Regressions include fixed effects for the geographic stratification unit and controls for the baseline level of the outcome variable. Standard errors are robust to clustering at the level of randomization (the village).

Table A5: Interaction: Head of household literate

(a) Treatment: CLTS

Bangladesh	(1) Ownership	(2) Usage (Men)	(3) Usage (Women)	(4) Any OD
Treatment	0.048*	0.081	0.025	-0.065
	(0.028)	(0.048)	(0.043)	(0.049)
HHH Literate	0.031	0.021	0.068	0.001
	(0.028)	(0.056)	(0.045)	(0.035)
Treatment X HHH Literate	-0.070	0.079	0.052	0.018
	(0.046)	(0.063)	(0.054)	(0.057)
Control group mean	0.071	0.586	0.652	0.617
Num. clusters	34	34	34	34
Num. households	809	750	774	777

Indonesia	(1) Ownership	(2) Usage (Men)	(3) Usage (Women)	(4) Any OD
Treatment	0.190	0.004	-0.106	-0.064
	(0.194)	(0.143)	(0.192)	(0.163)
HHH Literate	0.146*	0.042	-0.050	-0.050
	(0.077)	(0.082)	(0.153)	(0.091)
Treatment X HHH Literate	-0.190	0.005	0.115	0.049
	(0.198)	(0.142)	(0.195)	(0.161)
Control group mean	0.156	0.227	0.255	0.818
Num. clusters	152	152	152	152
Num. households	866	870	870	870

(Table continued next page.)

Table A5: Interaction: Head of household literate (continued)

(b) Treatment: CLTS + Subsidy

Bangladesh	(1) Ownership	(2) Usage (Men)	(3) Usage (Women)	(4) Any OD
Treatment	0.084***	0.142**	0.102**	-0.123**
	(0.024)	(0.055)	(0.043)	(0.052)
HHH Literate	0.029	0.018	0.063	0.010
	(0.028)	(0.054)	(0.045)	(0.036)
Treatment X HHH Literate	0.027	0.081	0.015	-0.066
	(0.042)	(0.065)	(0.052)	(0.057)
Control group mean	0.071	0.586	0.652	0.617
Num. clusters	74	74	74	74
Num. households	1119	1043	1073	1077

India	(1) Ownership	(2) Usage (Men)	(3) Usage (Women)	(4) Any OD
Treatment	0.224*	0.146	0.200*	-0.175**
	(0.117)	(0.099)	(0.103)	(0.082)
HHH Literate	0.063*	0.030	0.042	-0.057***
	(0.034)	(0.045)	(0.047)	(0.018)
Treatment X HHH Literate	-0.136	-0.067	-0.130	0.131*
	(0.116)	(0.091)	(0.096)	(0.075)
Control group mean	0.106	0.103	0.115	0.950
Num. clusters	79	79	79	79
Num. households	667	667	667	667

(Table continued next page.)

Table A5: Interaction: Head of household literate (continued)

(c) Treatment: Market Link

Bangladesh	(1) Ownership	(2) Usage (Men)	(3) Usage (Women)	(4) Any OD
Treatment	0.014	0.209***	0.093*	-0.191***
	(0.015)	(0.052)	(0.054)	(0.051)
HHH Literate	0.031	0.022	0.066	0.004
	(0.029)	(0.056)	(0.045)	(0.037)
Treatment X HHH Literate	0.034	-0.091	-0.115**	0.168**
	(0.047)	(0.062)	(0.055)	(0.073)
Control group mean	0.071	0.586	0.652	0.617
Num. clusters	32	32	32	32
Num. households	659	619	636	638

(d) Treatment: CLTS + Subsidy + Market Link

Bangladesh	(1) Ownership	(2) Usage (Men)	(3) Usage (Women)	(4) Any OD
Treatment	0.095***	0.150***	0.116**	-0.121***
	(0.024)	(0.052)	(0.046)	(0.045)
HHH Literate	0.029	0.018	0.065	0.008
	(0.028)	(0.053)	(0.044)	(0.036)
Treatment X HHH Literate	-0.002	0.009	-0.042	-0.043
	(0.041)	(0.058)	(0.049)	(0.052)
Control group mean	0.071	0.586	0.652	0.617
Num. clusters	69	69	69	69
Num. households	1134	1052	1090	1091

Notes: These tables display estimates of treatment interacted with an indicator for whether the head of the household is literate. This covariate was not available for Cambodia. The outcome variables (ownership, use, and open defecation) are as defined in the text and Appendix B. Results for Bangladesh control for the baseline level of the outcome variable of interest. Results for Bangladesh, Indonesia, and India include fixed effects for geographic units used in stratification. Standard errors are robust to clustering at the level of randomization (the village).

Table A6: Interaction: Household size

(a) Treatment: CLTS

Bangladesh	(1) Ownership	(2) Usage (Men)	(3) Usage (Women)	(4) Any OD
Treatment	0.029	0.106**	0.036	-0.071
	(0.022)	(0.050)	(0.031)	(0.043)
HH size	0.003	-0.024*	-0.011	0.021*
	(0.006)	(0.012)	(0.013)	(0.012)
Treatment X HH size	0.033*	0.023	0.020	-0.012
	(0.018)	(0.019)	(0.019)	(0.026)
Control group mean	0.068	0.518	0.583	0.646
Num. clusters	34	34	34	34
Num. households	1459	1361	1411	1414

Indonesia	(1) Ownership	(2) Usage (Men)	(3) Usage (Women)	(4) Any OD
Treatment	0.004	0.017	0.011	-0.018
	(0.025)	(0.031)	(0.031)	(0.029)
HH size	-0.002	-0.019	-0.017	0.016
	(0.016)	(0.018)	(0.018)	(0.016)
Treatment X HH size	0.008	0.016	0.015	-0.004
	(0.020)	(0.024)	(0.022)	(0.021)
Control group mean	0.154	0.221	0.247	0.822
Num. clusters	152	152	152	152
Num. households	915	919	919	919

(Table continued next page.)

Table A6: Interaction: Household size (continued)

(b) Treatment: CLTS + Subsidy

Bangladesh	(1) Ownership	(2) Usage (Men)	(3) Usage (Women)	(4) Any OD
Treatment	0.090***	0.181***	0.123***	-0.157***
	(0.020)	(0.039)	(0.027)	(0.034)
HH size	0.006	-0.022*	-0.011	0.017
	(0.007)	(0.012)	(0.014)	(0.012)
Treatment X HH size	0.006	0.018	0.014	0.020
	(0.010)	(0.017)	(0.017)	(0.015)
Control group mean	0.068	0.518	0.583	0.646
Num. clusters	75	75	75	75
Num. households	2082	1952	2010	2013

India	(1) Ownership	(2) Usage (Men)	(3) Usage (Women)	(4) Any OD
Treatment	0.111***	0.088***	0.093***	-0.047***
	(0.024)	(0.023)	(0.024)	(0.016)
HH size	0.007	0.006	0.007	-0.006
	(0.005)	(0.004)	(0.004)	(0.004)
Treatment X HH size	0.009	0.010	0.008	-0.007
	(0.007)	(0.008)	(0.008)	(0.007)
Control group mean	0.068	0.060	0.070	0.969
Num. clusters	80	80	80	80
Num. households	1433	1433	1433	1433

(Table continued next page.)

Table A6: Interaction: Household size (continued)

(c) Treatment: Market Link

Bangladesh	(1) Ownership	(2) Usage (Men)	(3) Usage (Women)	(4) Any OD
Treatment	-0.000	0.158**	0.031	-0.140***
	(0.014)	(0.061)	(0.055)	(0.047)
HH size	0.002	-0.025**	-0.008	0.018
	(0.006)	(0.012)	(0.013)	(0.012)
Treatment X HH size	-0.001	0.007	-0.008	0.024
	(0.010)	(0.019)	(0.015)	(0.023)
Control group mean	0.068	0.518	0.583	0.646
Num. clusters	32	32	32	32
Num. households	1218	1142	1180	1183

(d) Treatment: CLTS + Subsidy + Market Link

Bangladesh	(1) Ownership	(2) Usage (Men)	(3) Usage (Women)	(4) Any OD
Treatment	0.100***	0.175***	0.128***	-0.145***
	(0.016)	(0.035)	(0.029)	(0.033)
HH size	0.004	-0.021*	-0.010	0.018
	(0.006)	(0.012)	(0.014)	(0.012)
Treatment X HH size	0.008	0.006	0.010	-0.008
	(0.010)	(0.015)	(0.017)	(0.015)
Control group mean	0.068	0.518	0.583	0.646
Num. clusters	69	69	69	69
Num. households	2168	2028	2093	2098

(e) Treatment: Micro-Credit

Cambodia	(1) Ownership	(2) Usage	(3) Any OD
Treatment X HH size	0.006	0.044	-0.038
	(0.014)	(0.067)	(0.068)
Control group mean	0.279	0.196	0.804
Num. clusters	30	30	30
Num. households	1379	1383	1383

Notes: These tables display estimates of treatment interacted with household size (number of residents). This share is de-meaned (separately for each study) so that the level effect of treatment is the effect at the mean household size. The outcome variables (ownership, use and open defecation) are as defined in the text and Appendix B. For Cambodia, the ownership variable is an indicator for whether the household's willingness to pay in the BDM exercise was greater than or equal to USD 40 (in net present value for the financing arm). For use and OD, potential outcomes are simulated using the method described in section 6, with standard errors obtained from bootstrapping with replacement at the village level (500 replications). Results for Bangladesh control for the baseline level of the outcome variable of interest. Results for Bangladesh, Indonesia, and India include fixed effects for geographic units used in stratification. Standard errors are robust to clustering at the level of randomization (the village).

Table A7: Interaction: Household share women

(a) Treatment: CLTS

Bangladesh	(1)<br>Ownership	(2)<br>Usage (Men)	(3)<br>Usage (Women)	(4)<br>Any OD
Treatment	0.026	0.106**	0.033	-0.072
	(0.021)	(0.051)	(0.032)	(0.044)
HH share women	-0.014	0.062	-0.019	0.017
	(0.038)	(0.108)	(0.095)	(0.071)
Treatment X HH share women	0.058	-0.157	-0.093	0.059
	(0.085)	(0.116)	(0.115)	(0.135)
Control group mean	0.069	0.520	0.586	0.644
Num. clusters	34	34	34	34
Num. households	1450	1353	1402	1405

Indonesia	(1)<br>Ownership	(2)<br>Usage (Men)	(3)<br>Usage (Women)	(4)<br>Any OD
Treatment	0.005	0.014	0.009	-0.015
	(0.024)	(0.030)	(0.030)	(0.029)
HH share women	0.110	0.308***	0.255**	-0.252**
	(0.074)	(0.105)	(0.106)	(0.104)
Treatment X HH share women	-0.219*	-0.462***	-0.417***	0.356**
	(0.124)	(0.145)	(0.145)	(0.141)
Control group mean	0.154	0.221	0.247	0.822
Num. clusters	152	152	152	152
Num. households	915	919	919	919

(Table continued next page.)

Table A7: Interaction: Household share women (continued)

(b) Treatment: CLTS + Subsidy

Bangladesh	(1) Ownership	(2) Usage (Men)	(3) Usage (Women)	(4) Any OD
Treatment	0.089***	0.183***	0.122***	-0.156***
	(0.020)	(0.041)	(0.027)	(0.035)
HH share women	-0.014	0.069	-0.019	0.014
	(0.037)	(0.113)	(0.095)	(0.070)
Treatment X HH share women	0.005	-0.056	0.001	-0.032
	(0.064)	(0.138)	(0.128)	(0.094)
Control group mean	0.069	0.520	0.586	0.644
Num. clusters	75	75	75	75
Num. households	2070	1942	1999	2002

India	(1) Ownership	(2) Usage (Men)	(3) Usage (Women)	(4) Any OD
Treatment	0.112***	0.090***	0.094***	-0.048***
	(0.024)	(0.023)	(0.024)	(0.016)
HH share women	-0.024	-0.035	-0.040	-0.012
	(0.059)	(0.057)	(0.061)	(0.039)
Treatment X HH share women	0.201	0.256**	0.221**	0.033
	(0.127)	(0.107)	(0.110)	(0.067)
Control group mean	0.068	0.060	0.070	0.969
Num. clusters	80	80	80	80
Num. households	1433	1433	1433	1433

(Table continued next page.)

Table A7: Interaction: Household share women (continued)

(c) Treatment: Market Link

Bangladesh	(1) Ownership	(2) Usage (Men)	(3) Usage (Women)	(4) Any OD
Treatment	-0.004	0.156**	0.023	-0.133***
	(0.014)	(0.062)	(0.054)	(0.047)
HH share women	-0.021	0.060	-0.023	0.018
	(0.037)	(0.118)	(0.096)	(0.072)
Treatment X HH share women	0.066	0.075	0.250*	-0.102
	(0.058)	(0.187)	(0.126)	(0.139)
Control group mean	0.069	0.520	0.586	0.644
Num. clusters	32	32	32	32
Num. households	1209	1135	1172	1175

(d) Treatment: CLTS + Subsidy + Market Link

Bangladesh	(1) Ownership	(2) Usage (Men)	(3) Usage (Women)	(4) Any OD
Treatment	0.101***	0.174***	0.128***	-0.146***
	(0.016)	(0.037)	(0.029)	(0.034)
HH share women	-0.021	0.066	-0.020	0.015
	(0.037)	(0.113)	(0.095)	(0.072)
Treatment X HH share women	0.065	0.027	0.009	0.097
	(0.068)	(0.136)	(0.125)	(0.103)
Control group mean	0.069	0.520	0.586	0.644
Num. clusters	69	69	69	69
Num. households	2151	2018	2078	2083

(e) Treatment: Micro-Credit

Cambodia	(1) Ownership	(2) Usage	(3) Any OD
Treatment X HH share women	-0.184*	0.011	-0.025
	(0.107)	(0.065)	(0.068)
Control group mean	0.279	0.196	0.804
Num. clusters	30	30	30
Num. households	1379	1383	1383

Notes: These tables display estimates of treatment interacted with the share of household members who are adult women. This share is de-meaned (separately for each study) so that the level effect of treatment is the effect at the mean household share. The outcome variables (ownership, use, and open defecation) are as defined in the text and Appendix B. For Cambodia, the ownership variable is an indicator for whether the household's willingness to pay in the BDM exercise was greater than or equal to USD 40 (in net present value for the financing arm). For use and OD, potential outcomes are simulated using the method described in the text, with standard errors obtained from bootstrapping with replacement at the village level (500 repetitions). Results for Bangladesh control for the baseline level of the outcome variable of interest. Results for Bangladesh, Indonesia, and India include fixed effects for geographic units used in stratification. Standard errors are robust to clustering at the level of randomization (the village).

Table A8: Interaction: Household share children

(a) Treatment: CLTS

Bangladesh	(1) Ownership	(2) Usage (Men)	(3) Usage (Women)	(4) Any OD
Treatment	0.027	0.108**	0.033	-0.086*
	(0.021)	(0.052)	(0.032)	(0.043)
HH share children	-0.106	-0.157*	-0.096	0.901***
	(0.068)	(0.091)	(0.102)	(0.093)
Treatment X HH share children	0.033	0.153	0.089	0.111
	(0.098)	(0.171)	(0.149)	(0.143)
Control group mean	0.069	0.520	0.586	0.644
Num. clusters	34	34	34	34
Num. households	1450	1353	1402	1405

Indonesia	(1) Ownership	(2) Usage (Men)	(3) Usage (Women)	(4) Any OD
Treatment	0.005	0.015	0.010	-0.016
	(0.025)	(0.031)	(0.031)	(0.029)
HH share children	-0.017	0.372	0.301	-0.358
	(0.245)	(0.256)	(0.247)	(0.236)
Treatment X HH share children	-0.013	-0.205	-0.095	-0.042
	(0.341)	(0.336)	(0.327)	(0.322)
Control group mean	0.154	0.221	0.247	0.822
Num. clusters	152	152	152	152
Num. households	915	919	919	919

(Table continued next page.)

Table A8: Interaction: Household share children (continued)

(b) Treatment: CLTS + Subsidy

Bangladesh	(1) Ownership	(2) Usage (Men)	(3) Usage (Women)	(4) Any OD
Treatment	0.089***	0.183***	0.122***	-0.154***
	(0.020)	(0.041)	(0.028)	(0.036)
HH share children	-0.105	-0.157*	-0.094	0.895***
	(0.068)	(0.089)	(0.101)	(0.090)
Treatment X HH share children	0.137	0.176	0.128	-0.007
	(0.088)	(0.119)	(0.123)	(0.120)
Control group mean	0.069	0.520	0.586	0.644
Num. clusters	75	75	75	75
Num. households	2070	1942	1999	2002

India	(1) Ownership	(2) Usage (Men)	(3) Usage (Women)	(4) Any OD
Treatment	0.113***	0.090***	0.094***	-0.049***
	(0.024)	(0.024)	(0.024)	(0.016)
HH share children	-0.055	-0.055	-0.083	-0.012
	(0.069)	(0.058)	(0.060)	(0.038)
Treatment X HH share children	0.094	0.084	0.077	-0.037
	(0.126)	(0.109)	(0.115)	(0.073)
Control group mean	0.068	0.060	0.070	0.969
Num. clusters	80	80	80	80
Num. households	1433	1433	1433	1433

(Table continued next page.)

Table A8: Interaction: Household share children (continued)

(c) Treatment: Market Link

Bangladesh	(1) Ownership	(2) Usage (Men)	(3) Usage (Women)	(4) Any OD
Treatment	-0.003	0.158**	0.029	-0.150***
	(0.014)	(0.062)	(0.054)	(0.047)
HH share children	-0.105	-0.151	-0.089	0.895***
	(0.068)	(0.090)	(0.096)	(0.092)
Treatment X HH share children	0.038	0.055	-0.082	0.085
	(0.090)	(0.128)	(0.149)	(0.161)
Control group mean	0.069	0.520	0.586	0.644
Num. clusters	32	32	32	32
Num. households	1209	1135	1172	1175

(d) Treatment: CLTS + Subsidy + Market Link

Bangladesh	(1) Ownership	(2) Usage (Men)	(3) Usage (Women)	(4) Any OD
Treatment	0.101***	0.175***	0.128***	-0.148***
	(0.016)	(0.036)	(0.029)	(0.036)
HH share children	-0.108	-0.151*	-0.093	0.898***
	(0.067)	(0.090)	(0.101)	(0.091)
Treatment X HH share children	0.111	0.030	0.059	-0.029
	(0.090)	(0.117)	(0.123)	(0.130)
Control group mean	0.069	0.520	0.586	0.644
Num. clusters	69	69	69	69
Num. households	2151	2018	2078	2083

(e) Treatment: Micro-Credit

Cambodia	(1) Ownership	(2) Usage	(3) Any OD
Treatment X HH share children	0.142	-0.014	0.037
	(0.163)	(0.047)	(0.049)
Control group mean	0.279	0.196	0.804
Num. clusters	30	30	30
Num. households	1379	1383	1383

Notes: These tables display estimates of treatment interacted with the share of household members who are children under-5 years old. This share is de-meaned (separately for each study) so that the level effect of treatment is the effect at the mean household share. The outcome variables (ownership, use, and open defecation) are as defined in the text and Appendix B. For Cambodia, the ownership variable is an indicator for whether the household's willingness to pay in the BDM exercise was greater than or equal to USD 40 (in net present value for the financing arm). For use and OD, potential outcomes are simulated using the method described in the text, with standard errors obtained from bootstrapping with replacement at the village level (500 repetitions). Results for Bangladesh control for the baseline level of the outcome variable of interest. Results for Bangladesh, Indonesia, and India include fixed effects for geographic units used in stratification. Standard errors are robust to clustering at the level of randomization (the village).

Table A9: Interaction: Poverty indicator

(a) Treatment: CLTS

Bangladesh	(1) Ownership	(2) Usage (Men)	(3) Usage (Women)	(4) Any OD
Treatment	0.032	0.136**	0.065	-0.087
	(0.028)	(0.061)	(0.044)	(0.061)
Poverty Indicator	-0.040**	0.025	0.003	0.068*
	(0.017)	(0.050)	(0.046)	(0.039)
Treatment X Poverty Indicator	-0.007	-0.066	-0.059	0.031
	(0.033)	(0.057)	(0.057)	(0.058)
Control group mean	0.065	0.519	0.577	0.641
Num. clusters	34	34	34	34
Num. households	1590	1387	1516	1521

Indonesia	(1) Ownership	(2) Usage (Men)	(3) Usage (Women)	(4) Any OD
Treatment	0.014	0.030	0.021	-0.020
	(0.031)	(0.036)	(0.036)	(0.034)
Poverty Indicator	-0.037	-0.026	-0.051	0.032
	(0.034)	(0.035)	(0.033)	(0.035)
Treatment X Poverty Indicator	-0.039	-0.059	-0.052	0.024
	(0.049)	(0.050)	(0.049)	(0.050)
Control group mean	0.154	0.221	0.247	0.822
Num. clusters	152	152	152	152
Num. households	915	919	919	919

(Table continued next page.)

Table A9: Interaction: Poverty indicator (continued)

(b) Treatment: CLTS + Subsidy

Bangladesh	(1) Ownership	(2) Usage (Men)	(3) Usage (Women)	(4) Any OD
Treatment	0.106***	0.211***	0.151***	-0.186***
	(0.026)	(0.049)	(0.032)	(0.041)
Poverty Indicator	-0.033*	0.012	-0.013	0.072*
	(0.017)	(0.050)	(0.044)	(0.038)
Treatment X Poverty Indicator	-0.042	-0.057	-0.052	0.054
	(0.027)	(0.054)	(0.047)	(0.044)
Control group mean	0.065	0.519	0.577	0.641
Num. clusters	75	75	75	75
Num. households	2262	1984	2156	2163

India	(1) Ownership	(2) Usage (Men)	(3) Usage (Women)	(4) Any OD
Treatment	0.109***	0.100***	0.101***	-0.061**
	(0.033)	(0.029)	(0.030)	(0.024)
Poverty Indicator	-0.023	-0.007	-0.013	0.020
	(0.021)	(0.017)	(0.019)	(0.015)
Treatment X Poverty Indicator	0.023	-0.013	-0.004	0.016
	(0.041)	(0.035)	(0.036)	(0.030)
Control group mean	0.072	0.064	0.075	0.969
Num. clusters	80	80	80	80
Num. households	1250	1250	1250	1250

(Table continued next page.)

Table A9: Interaction: Poverty indicator (continued)

(c) Treatment: Market Link

Bangladesh	(1) Ownership	(2) Usage (Men)	(3) Usage (Women)	(4) Any OD
Treatment	0.032	0.223***	0.068	-0.208***
	(0.022)	(0.061)	(0.043)	(0.052)
Poverty Indicator	-0.042**	0.008	0.001	0.080**
	(0.018)	(0.050)	(0.046)	(0.039)
Treatment X Poverty Indicator	-0.049	-0.114	-0.046	0.099
	(0.029)	(0.075)	(0.056)	(0.061)
Control group mean	0.065	0.519	0.577	0.641
Num. clusters	32	32	32	32
Num. households	1322	1163	1269	1273

(d) Treatment: CLTS + Subsidy + Market Link

Bangladesh	(1) Ownership	(2) Usage (Men)	(3) Usage (Women)	(4) Any OD
Treatment	0.097***	0.187***	0.134***	-0.156***
	(0.021)	(0.046)	(0.034)	(0.042)
Poverty Indicator	-0.036*	0.017	-0.006	0.074*
	(0.018)	(0.050)	(0.044)	(0.038)
Treatment X Poverty Indicator	-0.000	-0.025	0.007	0.005
	(0.025)	(0.056)	(0.048)	(0.045)
Control group mean	0.065	0.519	0.577	0.641
Num. clusters	69	69	69	69
Num. households	2345	2058	2239	2247

(e) Treatment: Micro-Credit

Cambodia	(1) Ownership	(2) Usage	(3) Any OD
Treatment X Poverty Indicator	-0.013	0.021	-0.034
	(0.049)	(0.058)	(0.058)
Control group mean	0.279	0.196	0.804
Num. clusters	30	30	30
Num. households	1383	1383	1383

Notes: These tables display estimates of treatment interacted with an indicator for the household's poverty status. Each interaction is computed in a separate regression. The outcome variables (ownership, use, and open defecation) are as defined in the text and Appendix B. For Cambodia, the ownership variable is an indicator for whether the household's willingness to pay in the BDM exercise was greater than or equal to USD 40 (in net present value for the financing arm). For use and OD, potential outcomes are simulated using the method described in the text, with standard errors obtained from bootstrapping with replacement at the village level (500 repetitions). Results for Bangladesh control for the baseline level of the outcome variable of interest. Results for Bangladesh, Indonesia, and India include fixed effects for geographic units used in stratification. Standard errors are robust to clustering at the level of randomization (the village).

A.3 Experiment Design of Guiteras et al. (2015)

Figure A5: Design of Bangladesh Study

(a) Design of main treatments

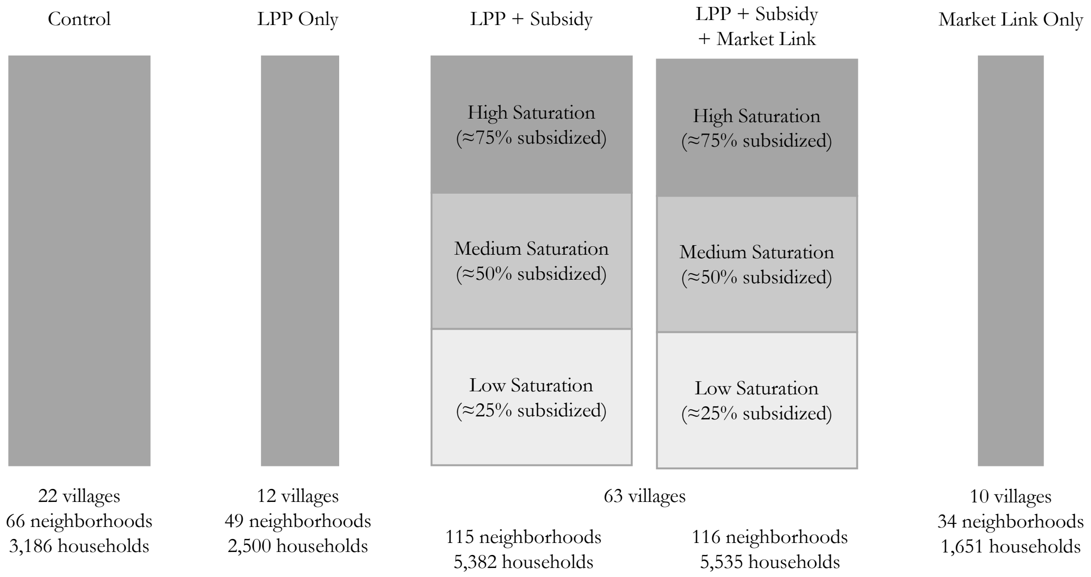

(b) Design of superstructure subsidy treatment

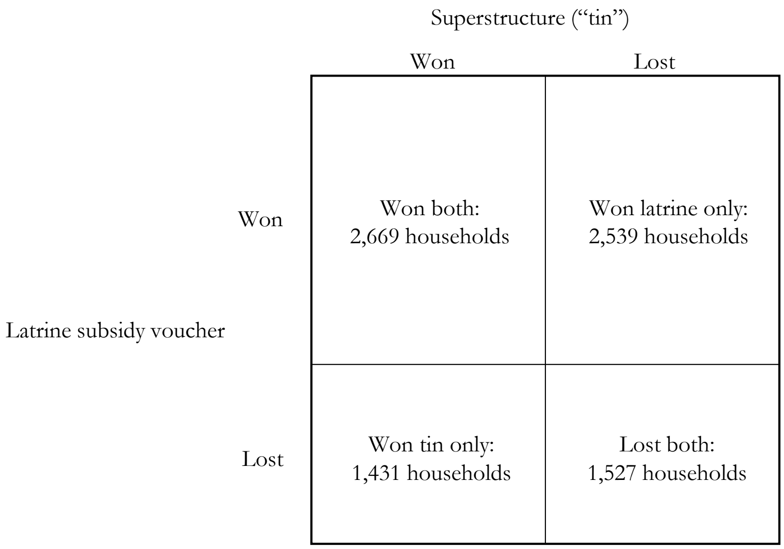

Notes: The sample sizes in the top figure encompass all households. The bottom figure shows the outcome of the two independent public lotteries in the LPP + Subsidy and LPP + Subsidy + Supply neighborhoods: one for a voucher for a subsidized latrine; the second for sheets of corrugated iron ("tin") to build a superstructure for a latrine. The areas of the rectangles are proportional to the share of households in each category. Total: 8,166 eligible households in subsidy villages (63 villages, 231 neighborhoods).

B Outcome Definitions and Measurement

In this appendix, we describe the steps to construct the different outcome variables of interest using data from the four interventions.

B.1 Bangladesh

Post-treatment outcome variable construction for Bangladesh is described as follows:

1. **Sanitation ownership:** We use the information on latrine usage from the follow-up survey, questions Q11 ("Where is the primary latrine located?") and Q12 ("What kind of toilet facility is it?"). The indicator takes value one if the response to Q11 takes value 01 (In own homestead) or 02 (outside own homestead, not attached). Furthermore, using Q12, we restrict the type of sanitation facility to suitable infrastructure types– i.e., we exclude option 01 (Don't have any latrine) and 02 (Hanging latrine) as suitable infrastructure.

2. **Sanitation usage:** Information on sanitation usage among adults is provided in questions Q3,4,5 (parts a and c) – which asks whether adult men (part a) and adult women (part c) in the household use a toilet shared with another household (Q3), a private toilet owned by household (Q4), a community toilet (Q5). The variable *Usage (Men)* is an indicator that takes value one if Q3.a, Q4.a, or Q5.a for adult men takes value one, and zero otherwise. Similarly, the variable *Usage (Women)* is an indicator that takes value one if Q3.c, Q4.c, or Q5.c for adult women takes value one, and zero otherwise.

3. **Any OD:** Lastly, information on the practice of open defecation within the household is provided in questions Q6.a, Q6.c., and Q6.e – which asks whether adult men (part a), adult women (part c), and children (part e) in the household use open spaces/bushes/hanging latrines (Q6). The variable *Any OD* is an indicator that takes value one if Q6.a, Q6.c, or Q6.e takes value one, and zero otherwise. In this case, we do not distinguish between men's and women's OD behavior and include children.

B.2 Cambodia

Because of the randomness in BDM, we need to construct synthetic purchase, installation, and usage variables that reflect what households (probabilistically) *would have done* at a given offer price. Post-treatment variable construction for Cambodia is described as follows:

1. **Purchase:** For a posited price P, we code all households as purchasing if their maximum WTP (as elicited in their BDM bid) was greater than or equal to P. Note some of these households, in fact, will have purchased (those with a draw less than or equal to their bid), and some will not have purchased (those with a draw greater than their bid), but all *would have purchased* at a fixed price of P. The main

price P that we consider is USD 40, corresponding to the rough break-even, or unsubsidized, cost of the latrine components.

2. **Sanitation usage:** We do not have information about male and female sanitation usage in Cambodia. In this case, we construct a single usage variable for both genders using QC.1 ("Do ADULTS (ages 18+) in your household use the latrine for defecation?"). The variable *Usage* is an indicator that takes value one if QC.1 takes the value 2 (Sometimes) or 3 (Almost always), and zero otherwise.

3. **Any OD:** Similarly, to capture improvement in sanitation behavior among members of the household regarding open defecation, we make use of questions C.1 ("Do adults (ages 18+) in your household use the latrine for defecation?") and C.2 ("Do children (ages 2-17) in your household use the latrine for defecation?"). The variable *Any OD* is an indicator that takes value one if QC.1 or QC.2 takes value 1 (Never or almost never), indicating that household members (neither adults nor children) never make use of the latrine facility.

B.3 India

Post-treatment variable construction for India is described as follows:

1. **Sanitation ownership:** We use the information on sanitation facilities from the follow-up survey, questions G.9.1 ("What is your household's main sanitation facility (main toilet facility)?") and G.9.9 ("Where is the toilet located"). The indicator takes value one if G.9.1 takes value one for one of the 1 through 9 options (as suitable sanitation infrastructure facilities). Furthermore, using G.9.9, we restrict the facility's location to options 01 and 02, i.e., the location of the toilet is either inside the household or in the household yard or land.

2. **Sanitation usage:** Information on sanitation usage among adults is provided in question G.9.23 ("Do household members practice open defecation?"). The indicator takes value one if G.9.23 takes value one for option 03 (Never) separately for adult men and adult women. We could not find a clean way to define sanitation usage, so we tried to back out the inverse of the OD variable for adults reflecting use. In addition, we construct separate variables for usage by adult men and adult women.

3. **Any OD:** Similarly, information on the practice of open defecation within the household is provided in question G.9.23 ("Do household members practice open defecation?"). The indicator takes value one if G.9.23 takes value one for option 01 (Daily) or 02 (Occasionally) for either men, women, or children. Unlike usage, we include children's OD practices in the definition. These restrictions are similar to the ones employed in Cameron et al. (2022) to ensure comparability.

B.4 Indonesia

Post-treatment variable construction for Indonesia is described as follows:

1. **Sanitation ownership:** We use the information on sanitation facilities from the follow-up survey, questions G9.1 ("Where do household members of your household usually do to defecate?"). The indicator takes value one if G9.1 takes value one for one of the 1 through 9 options (as suitable sanitation infrastructure facilities). Furthermore, using G9.9, we restrict the facility's location to options 01 and 02, i.e., the location of the toilet is either inside the household or in the household yard or land.

2. **Sanitation usage:** Information on sanitation usage among adults is provided in questions G.9.23 ("Do household members practice open defecation?"). The indicator takes value one if G.9.23 takes value one for option 03 (Never) separately for adult men and adult women. Similar to India, we were unable to find a clean way to define sanitation usage, so we tried to back out the inverse of the OD variable. In addition, we construct separate variables for usage by adult men and adult women.

3. **Any OD:** Information on the practice of open defecation within the household is provided in question G.9.23 ("Do household members practice open defecation?"). The indicator takes value one if G.9.23 takes value one for option 01 (Daily) or 02 (Occasionally) for either men, women, or children. Unlike usage, we include children's OD practices in our definition. These restrictions are similar to the ones employed in Cameron et al. (2022) to ensure comparability.

We note that our results on Indonesia differ from those in Cameron et al. (2019), due to the sample selection choices we made for compatibility with the other studies included in our analysis. To confirm this is the case, we restricted the sample to households present in baseline and endline, having a child under-5, and having non-missing height and weight for at least one child. In addition, following the sample selection choices in Cameron et al. (2019), we dropped entries with missing values for dirt floor presence, per capita income, poverty status, or baseline sanitation status. With these restrictions in place, we get similar counts in treatment and control and come reasonably close to matching most of the available summary stats. In particular, we match the household count within 1% and sanitation baseline summary stats within 1 pp in control and 4 pp in treatment. Our estimated treatment effects are within 10% of those reported in Cameron et al. (2019). We thank, without implicating, Lisa Cameron, Susan Olivia, and Manisha Shah for their guidance and advice with their data.
